# Phytochemical and antibacterial properties of calyces *Hibiscus sabdariffa* L.: an in vitro and in silico multitarget-mediated antibacterial study

**DOI:** 10.1186/s12906-025-04794-1

**Published:** 2025-02-18

**Authors:** Hend Khairy Fekry Ghaly, Fatema Aly Al-Yamany Younis, Azza Mahmoud Soliman, Sabha Mahmoud El-Sabbagh

**Affiliations:** 1 Botany and Microbiology Department, Faculty of Science, Menoufia University, Shebin El-Kom, Menoufia, Egypt; 2 Chemistry Department, Faculty of Science, Al-Azhar University (Girls Branch), Cairo, Egypt; 3 Biochemistry Department, Faculty of Science, Alexandria University, Alexandria, 21515 Egypt

**Keywords:** Roselle, GC-MS, Phytochemical screening, Antioxidant capacity, *A. Baumanii*, *E. Coli*, *K. pneumoniae*, *P. Aeruginosa*, Scanning electron microscopy, Antibacterial molecular docking simulations

## Abstract

**Background:**

Multidrug-resistant (MDR) bacteria pose a significant threat to human health worldwide by increasing the harmful impact of traditional synthetic antibiotics. Traditional medicinal plants have bioactive metabolites that can significantly modulate the growth rate, cell survival, and pathogenicity of antibiotic-resistant bacteria. *Hibiscus sabdariffa* L., known as Roselle or Karkade, belongs to the Malvaceae family. It is well-known for its edible aromatic red/purple calyces and is extensively utilized in the food industry and pharmacological applications. *H. sabdariffa* calyx bioactive phytocompounds have potent therapeutic activities such as antimicrobial, antidiabetic, antiobesity, antioxidant, anti-inflammatory, and anticancer properties.

**Methods:**

This study utilized gas chromatography-mass spectrometry (GC-MS) analysis to determine the volatile aromatic compounds that found in the hydroethanolic extract of *Hibiscus sabdariffa* calyces. The purpose was to verify the antibacterial properties of Roselle calyces against selective MDR clinical bacterial isolates, including *A. baumanii*,* E. coli*, *K. pneumoniae*, and *P. aeruginosa*.

**Results:**

The GC-MS spectrum profile revealed the presence of twenty-seven volatile organic components, including organic fatty acid derivatives, ester compounds, sugar derivatives, and terpene components. The major GC-MS fractionations and the main active chemical compositions of the hydroethanolic extract of *H. sabdariffa* flowers were (*E*)-10-Octadecenoic acid methyl ester (59.23%), 8,11-Octadecadienoic acid, methyl ester (11.51%), Butanedioic acid, 3-hydroxy-2,2-dimethyl-, diethyl ester (6.22%), Diethyl succinate/Butanedioic acid, diethyl ester (2.35%), and Heptadecanoic acid, 16-methyl-, methyl ester/Methyl isostearate (2.31%). The hydroethanolic extract of *H. sabdariffa* dried calyces demonstrated potent antibacterial properties (zones diameter of inhibition growth, MIC, MBC, and MBC/MIC) against selective MDR clinical bacterial isolates, such as A. *baumanii*, *E. coli*, *K. pneumoniae*, and *P. aeruginosa*, as determined by the phytochemical screening (TAC, TFC, and TPC) and antioxidant activity (DPPH). The surface morphological characteristics of the treated *A. baumanii*, *E. coli*, *K. pneumoniae*, and *P. aeruginosa* clinical isolates have been affected in comparison to the untreated forms by the hydroethanolic extract of *H. sabdariffa* calyces, as determined by scanning electron microscopy (SEM). In silico predictive investigation revealed that the volatile aromatic components of the hydroethanolic extract of Roselle calyces exhibited significant scoring functions, binding affinities, and non-covalent intermolecular interactions with the MenB lyase and DNA gyrase targets of *E. coli*. These interactions significantly enhanced the activities of the volatile aromatic components against the bacterial pathogenicity, cell survival, growth, and differentiation of selective MDR clinical bacterial isolates.

**Conclusions:**

According to the in vitro and in silico findings, the hydroethanolic extract of *H. sabdariffa* calyces has shown potentials as an effective antioxidant and antibacterial treatment. It contains volatile aromatic compounds that can modulate selective MDR Gram-negative clinical bacterial isolates.

**Supplementary Information:**

The online version contains supplementary material available at 10.1186/s12906-025-04794-1.

## Background

The multidrug-resistant (MDR) bacteria, also known as “ESKAPE” pathogens, include *E. faecium*, *S. aureus*, *K. pneumoniae*, *A. baumanii*, *P. aeruginosa*, and *Enterobacter* species. These bacteria cause numerous infectious diseases [1]. Identification of the MDR bacteria is based on their resistance to at least one antibacterial drug from ≥ 3 potent antibiotic classes, as determined by the in vitro antimicrobial susceptibility assay. The extensive drug-resistant (XDR) strains are characterized by their resistance to at least one antibacterial drug in all six antibiotic classes [2–4]. In order to address the proliferation of antibiotic-resistant strains and reduce the adverse effects of excessive antibiotic use, there is significant interest in exploring the potential of traditional medicinal plants and their bioactive phytochemicals for various antimicrobial applications [5, 6]. *Hibiscus sabdariffa* L., also known as Roselle or Karkade, is a versatile tropical plant that belongs to the Malvaceae family. It is a woody-based subshrub that is found in several Asian (Saudi Arabia, Malaysia, Indonesia, Thailand, China, and India) and African countries (Egypt, Sudan, Senegal, Tanzania, Nigeria, and Mali) [7–9]. As a perennial herb, the mature *H. sabdariffa* aromatic plant contains fresh, reddish, edible calyces with a unique taste and flavor [10, 11]. The bright-red calyces of *H. sabdariffa* L. contain several bioactive components such as anthocyanins, polyphenols, flavonoids, organic acids, alkaloids, terpenes, tannins, coumarins, carbohydrates, vitamins, minerals, glycosides, pectin, and mucilage. These compounds are widely used in producing soft drinks, teas, jellies, jams, preserves, sauces, wine, ice cream, desserts, and beverages [12–14]. Furthermore, these bioactive components are potentially associated with their numerous pharmaceutical and biological activities. These activities include antiparasitic, antifungal, antibacterial, antiviral, antioxidant, antispasmodic, antihypertensive, anti-inflammatory, anticancer, immunomodulatory, antidiabetic, anti-obesity, and hypocholesterolemic as well as cardio-, hepato-, nephro-, and neuroprotective effects [15–18]. Aromatic plants typically contain various volatile organic compounds such as acids, esters, alcohols, aldehydes, ketones, long-chain hydrocarbons, terpenes, furans, and phenols that potentially correlate with their various biological and pharmacological applications [19–21]. The acute-toxicological characteristics of the conventional synthetic antimicrobial medications limit their administration. Currently, promising multitarget-related antibacterial phytocompounds have been selected to reduce the adverse effects of the conventional synthetic reference drugs and to evaluate their restorative pharmacological properties against selective antibacterial potential targets [22]. Identifying the most promising targets for the putative ligands is considered a crucial step in drug design development. Molecular docking simulations aim to identify the novel therapeutic leads by predicting and visualizing their binding modes/poses and evaluating their scoring functions, binding affinities, and non-covalent intermolecular interactions toward selective and promising potential targets [23, 24]. The current study also utilized an in silico molecular docking analysis to assess the antibacterial properties of the main volatile aromatic components of the hydroethanolic extract of *H. sabdariffa* calyces as effective and promising multitarget-mediated antibacterial ligands against selective *E. coli* MenB lyase and DNA gyrase survival targets.

## Materials and methods

### Collection of *H. sabdariffa* calyx samples, chemicals, and reagents

The dried calyces of *H. sabdariffa* were taken from a local distributor in Damanhur, Al-Bohaira, Egypt that were freshly obtained from an Experimental Farm at the Faculty of Agriculture, Tanta University, Tanta, Egypt [25]. The seeds (Sabahia 17, dark-red color) of these calyces were previously identified by the Herbarium of the Medicinal and Aromatic Plants Research Farm, El-Kanater El-Khairia, El-Kalubia Governorate, Ministry of Agriculture, ARC, Giza, Egypt. Furthermore, we obtained an experimental approval from the Botany and Microbiology Department, Faculty of Science, Menoufia University, Shebin El-Kom, Menoufia, Egypt. The seeds of Roselle can readily cultivate in well-drained and/or poor soils (the Middle and the Upper Egypt clayey soils) with ∼ 65% humidity under drought and high temperatures (˃ 35 °C). In the cultivation process of Roselle, the organic fertilizers widely use to improve the growth rate, maturity time, plant yield and its phytochemical components, and the seed capabilities. The ideal NPK fertilizers include 68 Kg N, 32 kg P_2_O_5_, 24 kg K_2_O, and 4 L humic acid per fed. The organic fertilizers as 500 kg ammonium sulfate (100 kg N), 150 kg calcium superphosphate (22.5 kg P_2_O_5_), 50 kg potassium sulfate (24 kg K_2_O) as well as azotobacterine and phosphorein (biofertilizers) per fed are also used. The seeds of Roselle were cultivated in the second week of May and harvested in the third week of October in both seasons of 2022 and 2023, which were irrigated with 100 cubic meters per acre per 6–7 days to improve the phytochemical components of Roselle [26]. In the present study, HPLC-grade methanol and ethanol, dimethyl sulfoxide (DMSO), quercetin (95%), gallic acid (79.5%), Folin-Ciocalteu’s phenol reagent, sodium carbonate (NaCO_3_), aluminum chloride (AlCl_3_), ascorbic acid (vitamin C), and 1,1-diphenyl-2-picrylhydrazyl (≥ 95% DPPH) were supplied by Sigma-Aldrich (Merck, St. Louis, MO, USA). Other materials, chemicals, and reagents were an analytical grade (Merck) used as received. The ultrapure deionized water was produced by the Milli-Q synthesis system (Millipore Corp., Billerica, MA, USA). All solutions and buffers in this study were prepared using ultrapure deionized water.

### Extraction of calyx *H. sabdariffa* phytochemicals by microwave-mediated process

Prior to the in vitro study, the dried *H. sabdariffa* calyx samples were re-dried using a shade-drying process for two weeks at room temperature (25 °C) on a laboratory bench to reduce their moisture content. The re-dried Roselle samples were blended into a powdered form using a Panasonic Blender PANA-MX-801 S HG, Panasonic, Malaysia. The powdered form was preserved in a dark, air-tight polyethylene container and stored in a refrigerator (4 °C) until further usage. First, 100 g of the powdered form of *H. sabdariffa* calyces was soaked in a flask that contained 1000 mL of an aqueous ethanolic solution (1:10 *w/v*; 60–70% ethanol *v/v*), following a previously described method with minor modifications [16]. Subsequently, this flask was placed in the microwave cavity for 120 min at 70–90 °C and 400-W microwave power. After the extraction process was completed, the Roselle mixture was filtered using Whatman qualitative No.4 filter paper. The filtrated form was sterilized using a 22 μm pore size filter (Millipore, Molsheim, France). Then, this sterilized filtrate was evaporated and concentrated using a rotary vacuum evaporator (N-1000; Eyela, Tokyo, Japan) at 120 rpm, 70 °C, and 500 mbar. This was followed by the freeze-drying process using a lyophilizer (LGJ-12MC, Shin Lab Co., Korea). The extraction yield was 8.82 g of the hydroethanolic extract of Roselle per 100 g of the powdered form of *H. sabdariffa* dried calyces.

### Identification of calyx *H. sabdariffa* volatile aromatic components using GC-MS analysis

Separation and identification of volatiles of the hydroethanolic extract of *H. sabdariffa* calyces were done using Direct DB-1/5MS and TG-5MS fused silica capillary separating columns (30 m length, 0.25 mm inner diameter, and 0.25 μm film thickness) along with a trace GC1310-Ultra/ISQ mass spectrometer (Thermo Scientific, Austin, TX, USA). Roselle sample was initially injected on a 1:50 split mode at 250 °C. The oven temperature of the separating column was firstly maintained at 50 °C for 2 min and raised at an increasing rate of 5 °C/min to 230 °C held for 2 min. Moreover, a rate of 30 °C/min was added to the final temperature of 290 °C held for 2 min. Helium (He) was employed as a carrier gas with a constant flow-rate of 1.0 mL/min. The temperature of the ion-source M^+^ injector and the MS transfer line were maintained at 200 °C and 260 °C, respectively, for a total run time of 48 min. Using the Splitless mode of the GC and the Autosampler AS1300, a diluted sample of 1.0 µL was automatically injected for 30 s. The mass spectra profile was obtained using a full scan electron impact mode, specifically an electron ionization system, with ionization energy voltages of 70 eV. The scan range was from 10 to 400 *m/z*. The Roselle GC volatile aromatic fractionations were determined by comparing the Roselle volatile components’ retention time (RT) and mass spectra (*m/z*) to those in the WILEY 2009 and NIST 2011 v.2.3 mass spectral commercial libraries database. The relative retention times (RTs) of a series of *n-*alkanes (C_7_-C_36_) were used to calculate the relative retention indices (RIs) for all identified Roselle volatile compounds. All analyses were replicated twice. The GC–MS analyses were performed using a Perkin Elmer Clarus 500 gas chromatography (Shelton, CT, USA) coupled with a Perkin Elmer Clarus 500 MSD spectrometer (Shelton, CT, USA) [21].

### Phytochemical screening and antioxidant capacity of the hydroethanolic extract of *H. sabdariffa* calyces

#### Total anthocyanin content

The total anthocyanin content (TAC) of the hydroethanolic extract of Roselle calyces was determined using a V-5600 UV-Vis spectrophotometer and pH differential spectrophotometry. The pH differential method was utilized [27–29], with slight modifications. After diluting 0.5 mL Roselle samples with 4.5 mL buffer solutions of pH 1.0 and pH 4.5 to 5 mL, the absorbance was measured at 520 and 700 nm, respectively. Delphinidin-3-*O*-sambubioside is considered the most abundant anthocyanin in Roselle calyces. It has a molecular weight (MW) of 597.5 g/mol and a molar adsorption coefficient (*ε*) value of 23,800 L.mol^− 1^.cm^− 1^. In contrast, cyanidine-3-*O-*glucoside is considered the least abundant anthocyanin pigment in Roselle calyces, with a MW of 449.4 g/mol and a *ε* value of 26,900 L.mol^− 1^.cm^− 1^. All measurements were performed in triplicate. The results were expressed as mg delphinidin-3-*O*-sambubioside or cyanidin-3-*O*-glucoside equivalents of anthocyanins according to the following equation:

1 $$TAC = \left( \begin{gathered}\left[ {{{\left( {{A_{520}} - \,{A_{700}}} \right)}_{pH1.0}}\, - \,{{\left( {{A_{520}} - \,{A_{700}}} \right)}_{pH4.5}}} \right] \hfill \\*\,MW*\,DF\,*\,2 \hfill \\ \end{gathered} \right)/\varepsilon *\,I$$

Where **TAC** represents mg cyanidine-3-*O-*glucoside equivalents of anthocyanins/g of the dried form of the hydroethanolic extract of Roselle calyces, **MW** is the anthocyanin’s molecular weight, ***ε*** is the molar adsorption coefficient, **DF** is the dilution factor, and **I** represents the quartz cuvette path length (0.64 cm).

### Total flavonoid content

The total flavonoid content (TFC) of the hydroethanolic extract of *H. sabdariffa* calyces was evaluated using the AlCl_3_ colorimetric method [28, 30, 31], with minor modifications. Quercetin was used as a reference standard flavonoid at concentrations of 0.1, 0.5, 1, 2, 4, 6, 8, 10, 20, and 50 mg/mL. In a test tube, 0.5 mL of the hydroethanolic extract of *H. sabdariffa* calyces and 0.5 mL of 10% AlCl_3_ were thoroughly mixed and incubated in a dark place at room temperature (25 °C) for 60 min. The absorbance was recorded at 400–420 nm using a V-5600 UV-Vis spectrophotometer to measure the formation of a golden yellow color. All measurements were performed in triplicate. The TFC was estimated from the standard calibration curve of quercetin (*Y* = 1.231*x* + 0.015, *R*^*2*^ = 0.978). The result was expressed as mg quercetin equivalents (QUE) of flavonoid/g of freeze-dried hydroethanolic extract of *H. sabdariffa* calyces using the following equation:

2 $$\:{\mathbf{TFC}} = {\mathbf{Conc}}*1000/25$$

Where **Conc** represents the flavonoid concentration in the Roselle samples from the standard quercetin calibration curve (mg/mL).

### Total phenolic content

The total phenolic content (TPC) of the hydroethanolic extract of *H. sabdariffa* calyces was spectrophotometrically determined using the Folin-Ciocalteu method [32–34], with slight modifications. Gallic acid was used as a reference standard phenol at concentrations of 0.1, 0.5, 1, 2, 4, 6, 8, 10, 20, and 50 mg/mL. In a test tube, 0.1 mL of diluted Roselle extract (1:10) and 0.2 mL of diluted Folin-Ciocalteu’s phenol reagent (1:20) were mixed and vortexed. To develop a greenish-blue color, 0.5 mL of 20% Na_2_CO_3_ solution was added, and the mixture was then incubated under complete darkness conditions. After 120 min, the absorbance was determined at 765 nm against the control negative sample using a V-5600 UV-Vis spectrophotometer. All measurements were performed in triplicate. Using the standard calibration curve of gallic acid (*Y* = 0.116*x* + 0.087, *R*^*2*^ = 0.975), the TPC was estimated as mg gallic acid equivalents (GAE) of phenol/g of freeze-dried Roselle hydroethanolic extract and expressed using the following equation:

3 $$TPC = Conc*250*1000/25$$

Where **Conc** represents the phenol concentration in the Roselle samples, as determined by the standard gallic acid calibration curve (mg/mL).

### DPPH radical scavenging activity

The antioxidant activity of the hydroethanolic extract of *H. sabdariffa* calyces was determined using the DPPH free radical scavenging assay [35, 36], with slight modifications. In the presence of antioxidants, the stable purple-colored DPPH free radicals are rapidly neutralized and reduced to a yellow-colored and reduced DPPH form [7]. The changing of the optical density was recorded using a V-5600 UV-Vis spectrophotometer at 517 nm. The hydroethanolic extract of *H. sabdariffa* calyces was prepared at ten concentrations: 1, 5, 10, 20, 40, 60, 80, 100, 150, and 200 mg/mL. In a test tube, the total volume of the mixture was 500 𝜇L of Roselle samples, 125 𝜇L of DPPH solution (4 mg DPPH dissolved in 100 mL of 50% methanol), and 375 𝜇L of 50% methanol that mixed, vortexed, and incubated at 25 °C for 30 min. As a positive control, ascorbic acid as a reference standard antioxidant was used instead of Roselle samples. As a negative control, the total volume of the mixture was 125 𝜇L of DPPH solution and 875 𝜇L of 50% methanol. The change of color was determined at 517 nm using a V-5600 UV-Vis spectrophotometer. All measurements were performed in triplicate. The DPPH radical scavenging activity was expressed using the following equation:

4 $$\begin{gathered}DPPH{\text{ }}radical{\text{ }}scavenging{\text{ }}activity\left( \% \right) = \, \hfill \\\,\,\,\,\,\,\,\,\,\,\,Ab{s_{control}} - \left( {Ab{s_{sample}}\,/\,Ab{s_{control}}} \right)\,*\,100 \hfill \\ \end{gathered} $$

Where **Abs**_**control**_ is the absorbance of negative control, and **Abs**_**sample**_ is the absorbance of Roselle samples or positive control (ascorbic acid) [35].

The antioxidant capacity of Roselle was expressed as a DPPH radical IC_50_ value (mg/mL) compared to ascorbic acid. Based on the linear regression equation, the DPPH radical IC_50_ value represented the concentration of Roselle (mg/mL) that inhibited 50% of DPPH free radicals compared to ascorbic acid (Fig. S6, Tables S2 and S3).

### Antibacterial activities of the hydroethanolic extract of *H. sabdariffa* calyces against selective MDR clinical bacterial isolates

#### Collection, isolation, and purification of MDR clinical bacterial isolates

Clinical isolates of the urinary tract (urine), wounds (pus), and respiratory tract (sputum) infections were extensively administered for selective pathogenic bacteria. The selected isolates were carefully collected, preliminary identified, isolated, and purified from patients that correlated to Alexandria University Hospitals, Faculty of Medicine, Alexandria, Egypt, from January to August 2022. The selective pathogenic Gram-negative (GN) bacteria were *A. baumanii*,* E. coli*, *K. pneumoniae*, and *P. aeruginosa*. Before processing, the purified specimens were quickly transported under aseptic conditions to the Bacteriology Lab, Damanhur Regional Joint P.H. Lab, Health Affairs Directorate, Damanhur, Al-Bohaira governorate, Egypt. This study was approved by the Ethics Committee and an Institutional Review Board (IRB) of Alexandria University Hospitals, Alexandria, Egypt.

#### Bacterial identification and antimicrobial susceptibility testing

Colony samples from primary cultures were subjected to cultural, morphological, and biochemical tests to verify the identification and characterization of specific clinical bacterial isolates [37]. The purified bacterial colonies were identified using an accurate and rapid direct inoculation method. The method was performed using the full automated colorimetric VITEK^®^-2 GN Cassette and N-280/AST panel Compact Systems according to the manufacturer’s recommendations (Biomérieux, Marcy-l’E´ toile, France). The VITEK^®^-2 test cards were employed to identify Gram-negative rods (GNRs) through using the Gram-negative Cassette [38]. The probability was 99% *A. baumanii*, 96% *E. coli*, 98% *K. pneumoniae*, and 99% *P. aeruginosa*. The antimicrobial susceptibility testing (AST) was conducted on selective MDR clinical bacterial isolates using multiple standard antibiotics (Oxoid Ltd., UK). The testing was performed on plate, nutrient or Mueller–Hinton agar (MHA) media using the agar-disc diffusion method. The interpretation criteria based on the guidelines that provided by the Clinical and Laboratory Standards Institute (CLSI) as bacterial susceptibility (S), moderate susceptibility (I), and bacterial resistance (R) (Table S1, Fig. S4A-D) [39]. According to the in vitro antimicrobial susceptibility assay, the MDR bacteria are identified according to their resistance (non-susceptibility) for at least one standard antibacterial drug from ≥ 3 potent antibiotic classes. While, the XDR strains are termed resistant to all standard antibiotics [3, 4].

### Antibacterial activity assay

The clinical bacterial isolates were cultivated in nutrient or Mueller-Hinton broth media for 24 h at a temperature of 37 °C until reaching a turbidity level of 0.5 on the McFarland standards scale. The inoculum size used was 1.5 × 10^8^ colony-forming units (CFU)/mL. In order to perform the agar-disc diffusion method, these inoculated suspensions were carefully swabbed onto the surface of MHA (Oxoid Ltd, UK) plates using sterile cotton swabs and allowed to dry. Sterile filter paper discs (5–6 mm; Difco, Detroit, MI, USA) were saturated in triplicates with 25 µL of serial dilution of tigecycline (TGC) as a positive control (4, 2, 1.2, 0.6, 0.2, 0.04, and 0.02 mg/mL). Alternatively, 50 µL of Roselle calyx samples (10, 6, 4, 2, 1, 0.5, and 0.1 mg/mL) were placed on the surface of each inoculated plate for each MDR bacterial isolate. As a negative control, 50 µL of 10% DMSO was used. Subsequently, the labeled cultured petri plates were incubated at 37 °C for 24 h. The antibacterial effectiveness of the hydroethanolic extract of *H. sabdariffa* calyces was assessed by measuring the zone diameters of inhibition bacterial growth (mm), minimum inhibitory concentrations (MICs), minimum bactericidal concentrations (MBCs), and MBC/MIC ratios. These measurements were compared to those of TGC that served as a positive control, following the CLSI guidelines [39]. The measurements were conducted three times. The zones of inhibition observed in bacterial growth indicate the absence of bacterial growth due to the inhibitory properties of Roselle concentrations that diffused into semisolid culture agar media under the Roselle-saturated discs. The study conducted by Abu Eleneen et al. assessed the diameter of inhibition zones for bacterial growth using the hydroethanolic extract of *H. sabdariffa* calyces. The criteria for evaluation were as follows: zone ≤ 9 mm (bacterial resistance), 10–15 mm (moderate resistance), and ≥ 16 mm (bacterial susceptibility) [4]. MIC refers to the lowest concentration of the diluted hydroethanolic extract of *H. sabdariffa* calyces that inhibited any visible growth of the tested bacterial isolates after incubation at 37 °C for 24 h [2, 40]. In contrast, the MBC of the diluted hydroethanolic extract of *H. sabdariffa* calyces was found to be the lowest concentration that completely inhibited the growth of the tested bacterial isolates after incubation at 37 °C for 48 h [37, 41, 42]. The MBC/MIC ratio is ˂ 4, demonstrating the bactericidal characteristics, while it is ≥ 4, reflecting the bacteriostatic activities of the selective potential antimicrobial drugs [43, 44].

### Scanning electron microscopy (SEM) examination

The scanning electron microscope (SEM) was used to evaluate the morphological changes that presented on the surface of the treated clinical isolates of A. *baumanii*, *E. coli*, *K. pneumoniae*, and *P. aeruginosa* with the hydroethanolic extract of *H. sabdariffa* L. calyces compared with the untreated forms. The selected samples were coated with gold and dried using a series of ethyl alcohol. Then, the preserved specimens were examined under SEM (JSM 1400 PLUS-JEOL, Tokyo, Japan).

### Molecular docking simulations analysis

Molecular docking is a significant computational-based method used to generate the conformations and orientations of ligands into the active-binding sites of their potential targets. Ranked poses were generated by search algorithms based on their scoring functions [45, 46]. In order to evaluate the effectiveness of selective promising volatile aromatic components of the hydroethanolic extract of *H. sabdariffa* calyces as potent antibacterials, the molecular docking simulations analysis was carried out between (*E*)-10-Octadecenoic acid methyl ester (MF: C_19_H_36_O_2_, MW:296.5 g/mol, CID:5364425), Butanedioic acid, 3-hydroxy-2,2-dimethyl-, diethyl ester (MF: C_10_H_18_O_5_, MW:218.25 g/mol, CID:589302), or Diethyl succinate (MF: C_8_H_14_O_4,_ MW:174.19 g/mol, CID:31249) with the X-ray crystallographical structures of *E. coli* K-12 1,4-Dihydroxy-2-naphthoyl-CoA synthase (MenB, lyase, EC: 4.1.3.36, PDB DOI: 3T88, 2.00Å) [47] and *E. coli* K-12 DNA Gyrase-DNA binding and cleavage domain in State1 without TOPRIM insertion (DNA gyrA and B subunits, isomerase, EC: 5.6.2.2, PDB DOI: 6RKS, 4.00Å) [48].

**Fig. 1:**
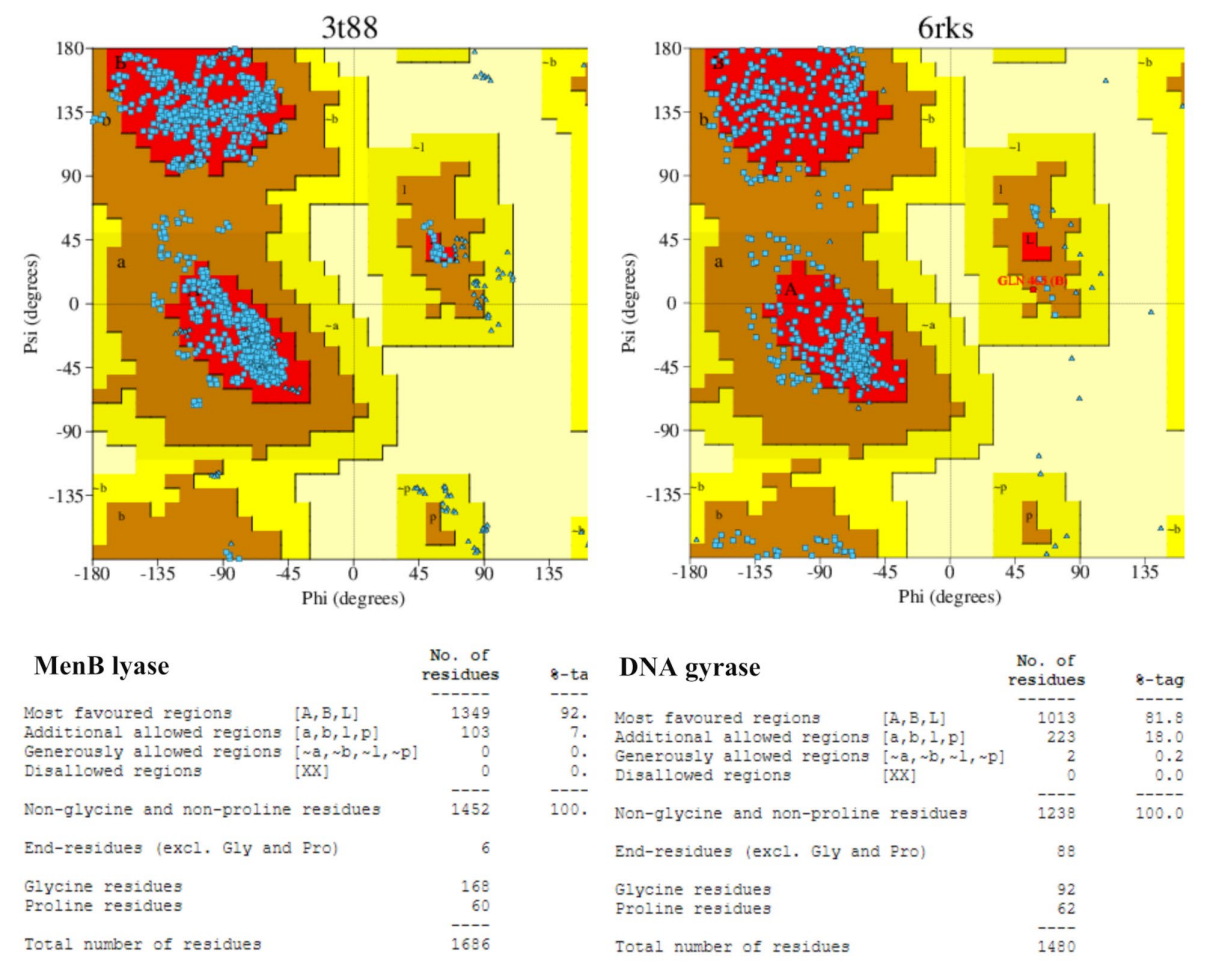
The Ramachandran plots and their statistics represent the good-quality stabilized models for selective promising antibacterial-related survival proteins (https://www.ebi.ac.uk/thornton-srv/software/PROCHECK/*).* Most favored regions (A, B, L) as red color; Additional allowed regions (a, b, l, p) as brown color; Generously allowed regions (∼ a, ∼b, ∼l, ∼p) as bright-yellow color; Disallowed regions (XX) as light-yellow color. The selected antibacterial-related survival targets were demonstrated as the following: *E. coli* K-12 1,4-Dihydroxy-2-naphthoyl-CoA synthase (MenB, lyase, 3T88) and *E. coli* K-12 DNA Gyrase-DNA binding and cleavage domain in State1 without TOPRIM insertion (DNA gyrA and B subunits, isomerase, 6RKS)

The protein structure files (PDB-file) were obtained from the RCSB-PDB database server, accessed on 5 March 2024 (http://www.rcsb.org/). The canonical SMILES of (*E*)-10-Octadecenoic acid methyl ester, Butanedioic acid, 3-hydroxy-2,2-dimethyl-, diethyl ester, and Diethyl succinate were retrieved from the PubChem database server (https://pubchem.ncbi.nlm.nih.gov/). Then, their 2D-chemical structures were generated and cleaned using ACD/ChemSketch software as.mol format files. The cleaned 2D-chemical structures of Roselle main volatile components were converted to.pdb format files using OpenBabel v2.3.2 software. The Swiss-PDBViewer v4.1.0 program was utilized to minimize the energy of target proteins. The protein models’ quality was validated using the Ramachandran plot study through the PROCHECK/PDBsum database server (https://www.ebi.ac.uk/thornton-srv/software/PROCHECK/*)* [49]. The Ramachandran plots and their statistics for the selected antibacterial-correlated targets are demonstrated in Fig. 1. The Ramachandran plot statistics indicate that 92.9% and 81.8% of the amino acid residues in the MenB lyase and DNA gyrase targets, respectively, are in the most favored regions, as shown in Fig. 1.

The molecular docking simulations tool Autodock 4.2.6 was used to investigate the estimated ligand-protein free energy of binding (kcal/mol), estimated inhibition constant (*Ki*), and the stability root-mean-square deviation-tolerance (RMSD-tol) values. The grid box properties were 0.375Å spacing, -27.419X-, 36.819Y-, and − 24.954Z-center, and 200 as a number of points in X-, Y-, and Z-dimensions. Free energy of binding was estimated to be less than − 5 kcal/mol, indicating that the protein target exhibits a specific binding affinity towards the lead [50–53]. The estimated free energy of binding for the docked ligands is positively correlated with their binding affinities and docking properties toward selective promising targets. The main Roselle volatile components with the highest binding energies/the lowest negative values toward *E. coli* MenB lyase and DNA gyrase were chosen after conducting molecular docking simulations. After molecular docking simulations, the main Roselle volatile components with the highest binding energies/the lowest negative values toward *E. coli* MenB lyase and DNA gyrase were selected to visualize their docked forms using the BIOVIA Drug Discovery Studio Visualizer software (Figs. 6-9).

**Fig. 2:**
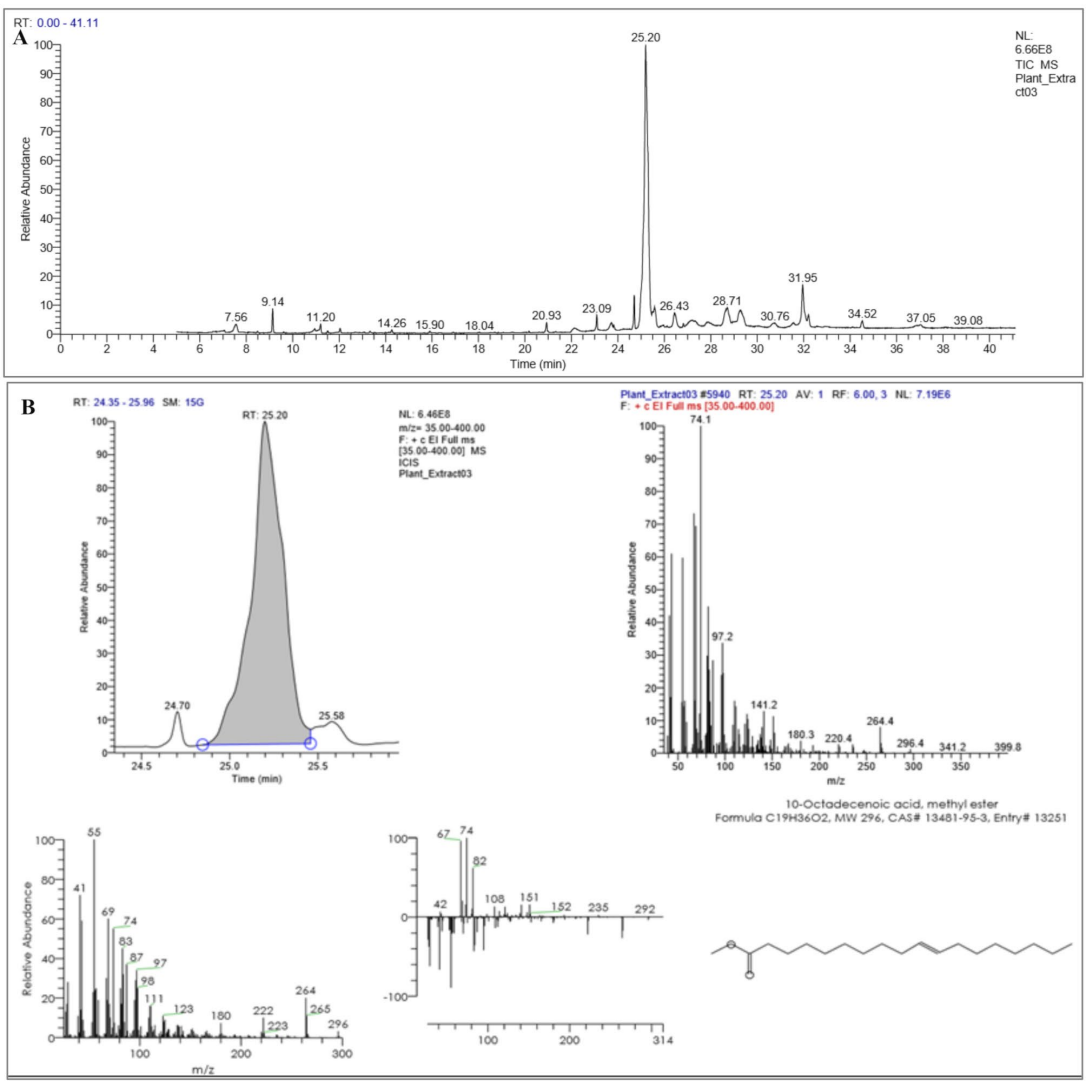
(**A**) The GC-MS chromatogram of twenty-seven fractionations/volatile organic compounds of the hydroethanolic extract of *H. sabdariffa* calyces/flowers. (**B**) The GC-MS analysis plot, mass spectrum, and 2D-molecular structure of (*E*)-10-Octadecenoic acid methyl ester (25.20 RT; 59.23%) that represented the most abundant fraction and the major active ingredient in the hydroethanolic extract of *H. sabdariffa* flowers

**Fig. 3:**
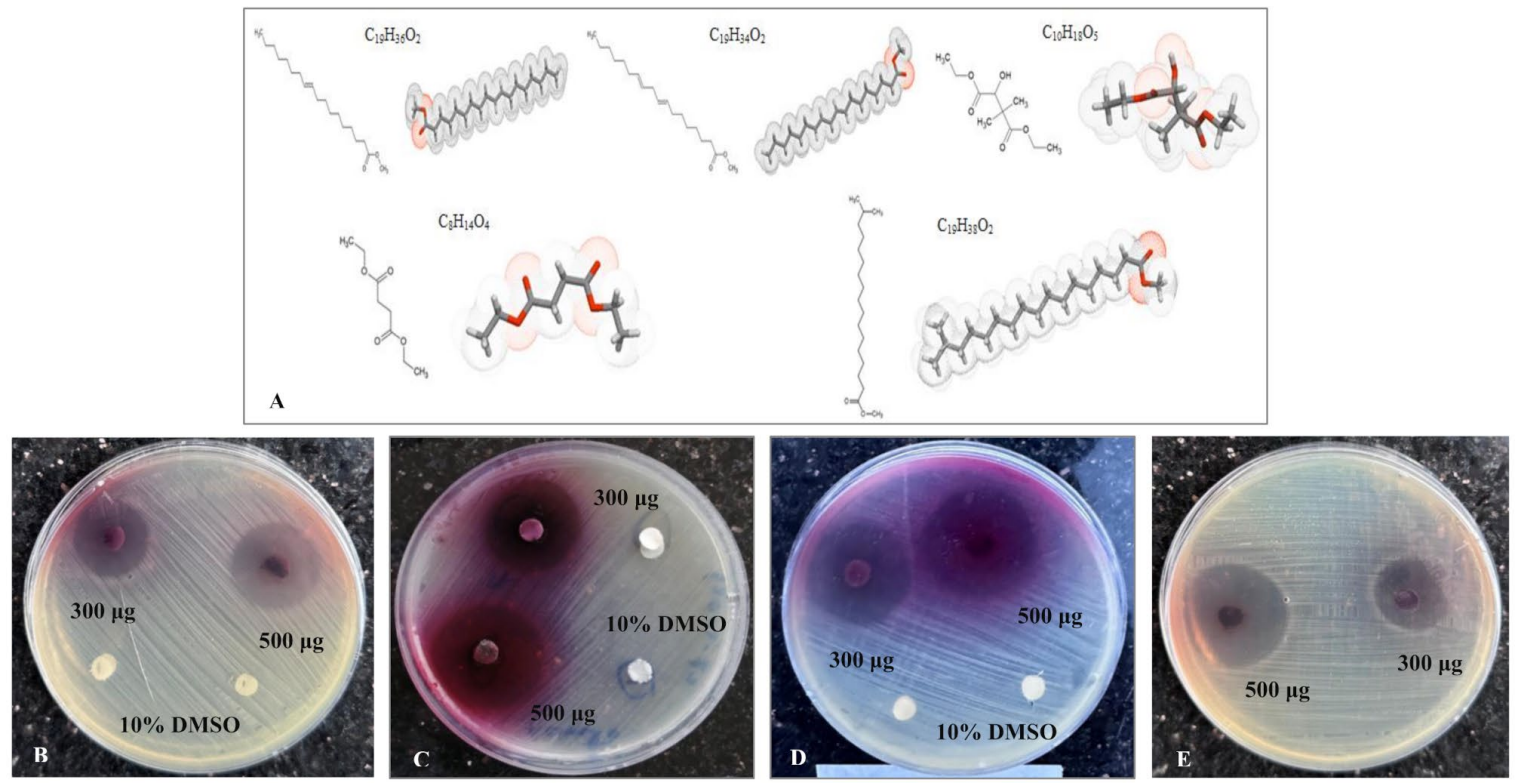
(**A**) The 2D- and 3D-molecular structures of the major volatile organic compounds in the hydroethanolic extract of *H. sabdariffa* calyces were (*E*)-10-Octadecenoic acid methyl ester (25.20 RT and 59.23%; C_19_H_36_O_2_), 8,11-Octadecadienoic acid, methyl ester (28.71 RT and 4.27%, 29.28 RT and 4.17%, and 25.58 RT and 3.06% fractionations; C_19_H_34_O_2_), Butanedioic acid, 3-hydroxy-2,2-dimethyl-, diethyl ester (31.96 RT and 6.22%; C_10_H_18_O_5_), Diethyl succinate/Butanedioic acid, diethyl ester (24.70 RT and 1.68% and 20.93 RT and 0.67% fractionations; C_8_H_14_O_4_), and Heptadecanoic acid, 16-methyl-, methyl ester/Methyl isostearate (26.44 RT and 2.31%; C_19_H_38_O_2_). Zone diameters of inhibition growth (mm) of 10 (500 µg/disc) and 6 (300 µg/disc) mg/mL hydroethanolic extract of *H. sabdariffa* calyces toward clinical *A. baumanii* (**B**), *E. coli* (**C**), *K. pneumoniae* (**D**), and *P. aeruginosa* (**E**) isolates compared to 10% DMSO as a negative control

**Fig. 4:**
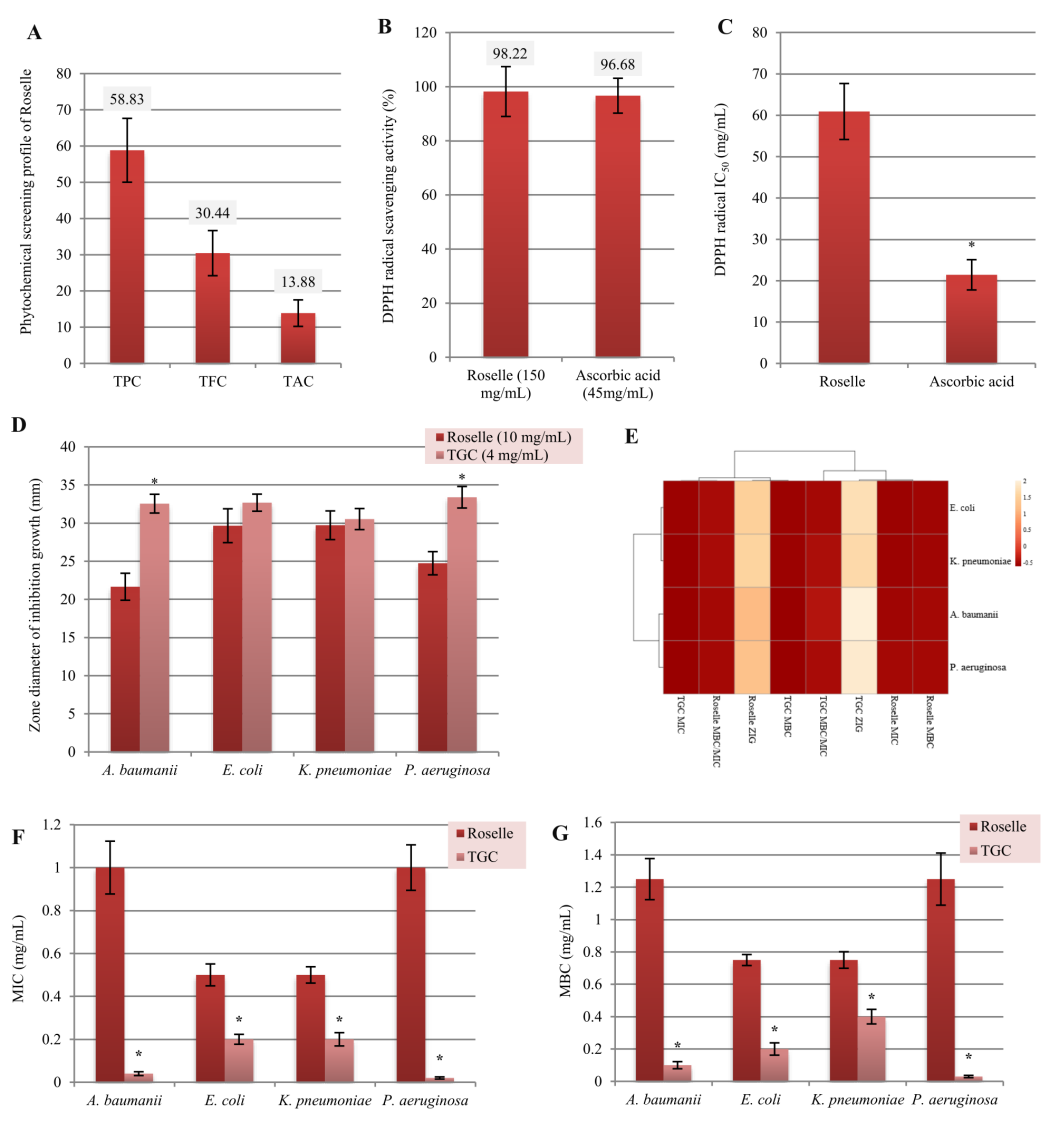
Phytochemical screening (TPC, TFC, and TAC) (**A**), DPPH radical scavenging activity (%) (**B**), DPPH radical IC_50_ value (**C**), zone diameters of inhibition growth (mm) (**D**), ClustVis heatmap distribution analysis (**E**), MIC (**F**), and MBC (**G**) of the hydroethanolic extract of *H. sabdariffa* calyces toward clinical *A. baumanii*,* E. coli*, *K. pneumoniae*, and *P. aeruginosa* isolates. Ascorbic acid and TGC were used as a reference standard antioxidant compound and the most effective antibiotic against these clinical MDR bacterial isolates, respectively. Data values are expressed as means ± S.D. (*n* = 3). * indicates statistically significant differences (*p* < 0.05) compared to ascorbic acid as a standard antioxidant or TGC as a reference standard antibiotic. ZIG, Zones of inhibition bacterial growth (mm)

**Fig. 5:**
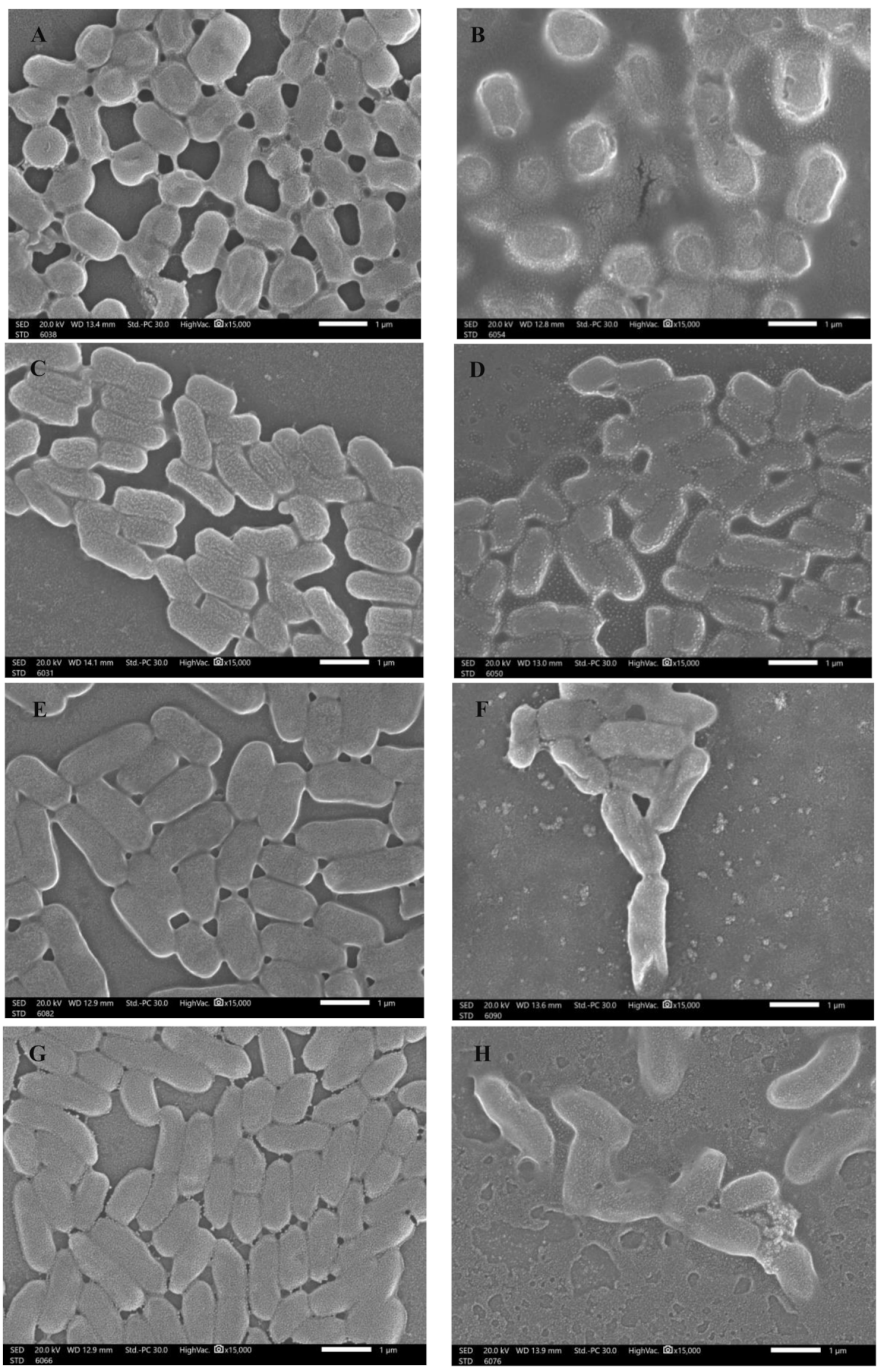
The surface morphological characteristics (SEM) of the untreated (**A**, **C**, **E**, and **G**) and treated (**B**, **D**, **F**, and **H**) *A. baumanii*,* E. coli*, *K. pneumoniae*, and *P. aeruginosa* clinical isolates, respectively with the hydroethanolic extract of *H. sabdariffa* calyces were demonstrated. Magnifications = 15,000x. Scale bar = 1 µM

### Statistical analysis

All data are statistically expressed as means ± standard deviations of the means (means ± S.D.) for in vitro study. The paired-sample test analysis of SPSS Windows Version 19.0 (SPSS, Inc., Chicago, IL, USA) was utilized to assess the statistical significance (*p* < 0.05) of the differences [54]. Compared to ascorbic acid, the linear regression analysis revealed that the roselle hydroethanolic extract exhibited a 50% inhibition (IC50) against DPPH free radicals. The ClustVis web server applications are freely available at http://biit.cs.ut.ee/clustvis/ server and were utilized to summarize and visualize the data values in an ideal heatmap distribution form [55].

## Results and discussion

### Identified the extract of *Hibiscus sabdariffa* L. calyces using GC-MS 

*Hibiscus sabdariffa* L. is an edible medicinal aromatic plant extensively used in food, cosmetics, and pharmaceutical industries. Roselle calyces possess potent therapeutic properties such as antioxidant, anti-inflammatory, antimicrobial, antiviral, antidiabetic, and anticancer that are significantly correlated with their active phytochemicals [32]. The medicinal aromatic plants contain numerous volatile organic components such as acids, esters, alcohols, aldehydes, ketones, long-chain hydrocarbons, terpenes, furans, and phenols that potentially associate with their different biological and pharmacological applications [19–21].

The hydroethanolic extract of *H. sabdariffa* calyces (Egypt origin) was analyzed using GC–MS screening assay to identify the volatile organic compounds. This was done to validate the pharmacological potentials of Roselle flowers against selective MDR clinical bacterial isolates. The prediction of phytocompounds was correlated with the WILEY and the National Institute Standards and Technology (NIST) commercial libraries database. The MS spectra profile revealed **twenty-seven** GC fractionations (volatile chemical compounds) that were identified in the hydroethanolic extract of *H. sabdariffa* flowers at the retention time (RT) from 7.56 to 37.03 (Tables 1 and 2; Fig. 2A). These volatile organic components were identified as organic fatty acid derivatives, alcohols, ester compounds, sugar derivatives, furans, and terpene components. The MS spectra and 2D-molecular structures of the major **five** active aromatic components of the hydroethanolic extract of *H. sabdariffa* flowers were clearly demonstrated in Figs. 2B and 3A, and Figs. S1-S3. The main **five** volatile components were (*E*)-10-Octadecenoic acid, methyl ester (59.23%), 8,11-Octadecadienoic acid, methyl ester (11.51%), Butanedioic acid, 3-hydroxy-2,2-dimethyl, diethyl ester (6.22%), Diethyl succinate/Butanedioic acid, diethyl ester (2.35%), and Heptadecanoic acid, 16-methyl-, methyl ester/Methyl isostearate (2.31%). These main volatiles represent 81.62% of the total volatile aromatic constuients of the hydroethanolic extract of *H. sabdariffa* calyces (Tables 1 and 2; Fig. 2A), and have potent antioxidant, anti-inflammatory, antibacterial, antifungal, anticancer, and hypolipidemic characteristics [56–59]. In this study, the restorative pharmacological characteristics of Roselle calyces potentially correlate with the main volatile components that clearly reported in the Roselle calyces’ GC-MS analysis profile (Tables 1 and 2). The presented findings in the *H. sabdariffa* flowers’ GC-MS analysis profile were matched in this study with previous Roselle calyces’ GC-MS phytochemical screening that clearly reflect the relationship between the antioxidant, anti-inflammatory, anticancer, and antimicrobial activities of Roselle calyces and their bioactive volatile components [3, 5, 8, 10, 15, 17, 60, 61]. The GC-MS screening profile of the hydroethanolic extract of Roselle calyces (Indonesia origin) demonstrate **seventeen** volatile organic components, including (2*E*)-5-Methyl, [2,3*-D*2] hexa-2,4-dieonic acid, Propanoic acid, 2-Furancarboxaldehyde, 5-(Hydroxymethyl)-, *n*-Hexadecanoic acid, and (9*E*)-9-Octadeconoic acid that mainly correlate with the dental antibacterial activities of Roselle calyces [62].

**Fig. 6:**
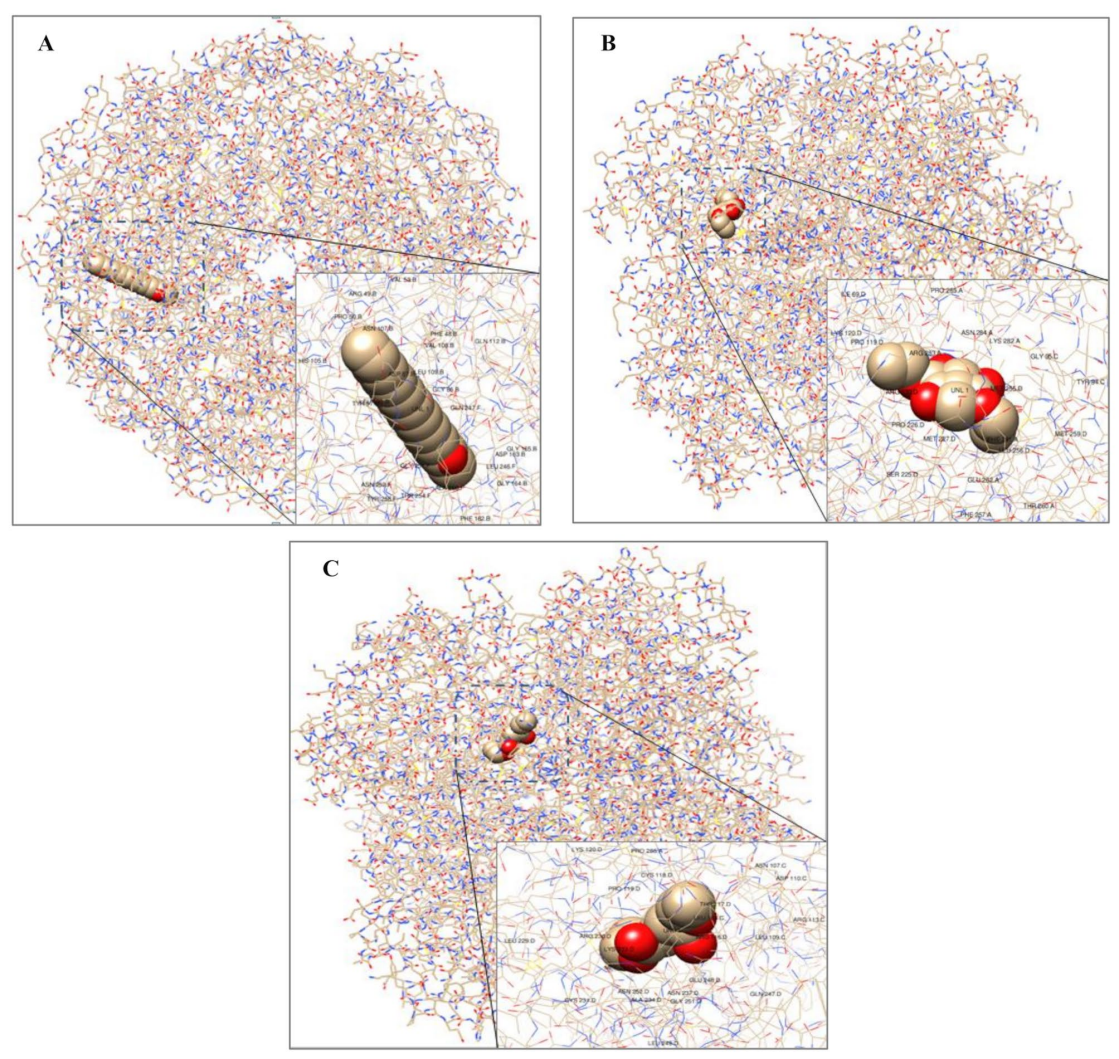
The 3D-prepared structures of the highest binding mode of (*E*)-10-Octadecenoic acid methyl ester (**A**), Butanedioic acid, 3-hydroxy-2,2-dimethyl-, diethyl ester (**B**), and Diethyl succinate (**C**) toward selective promising antibacterial-correlated target as *E. coli* MenB lyase (3T88)

**Fig. 7:**
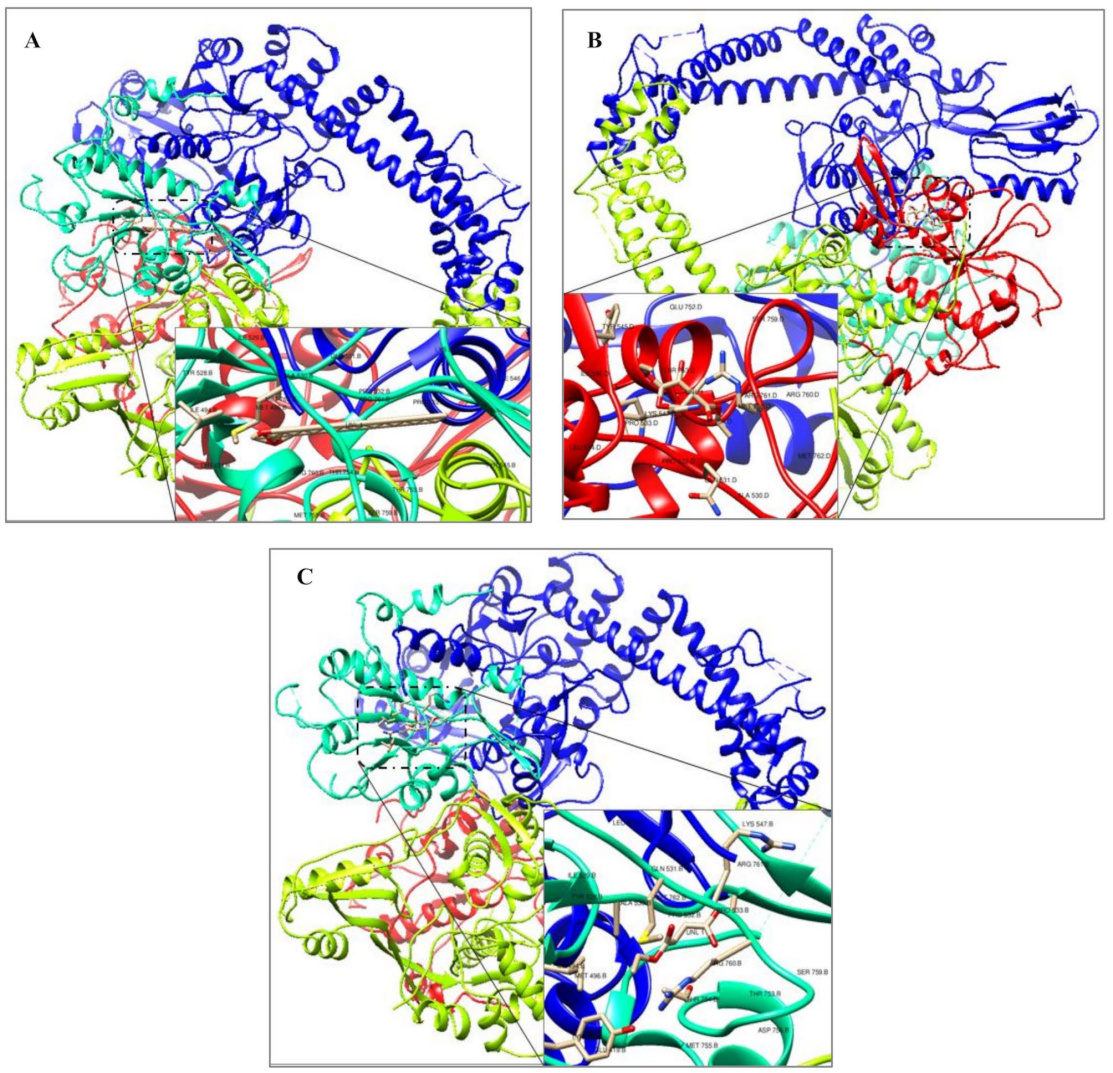
The 3D-prepared structures of the highest binding mode of (*E*)-10-Octadecenoic acid methyl ester (**A**), Butanedioic acid, 3-hydroxy-2,2-dimethyl-, diethyl ester (**B**), and Diethyl succinate (**C**) toward selective promising antibacterial-correlated target as *E. coli* DNA gyrase (6RKS)

### Phytochemical profile and antioxidant activity of the extract of *H. sabdariffa* calyces

Roselle calyces are considered an abundant source of several bioactive aromatic components, such as organic acids, anthocyanins, terpenes, furans, polyphenols, and flavonoids. These components are considerably associated with the antioxidant, antimicrobial, anti-inflammatory, antidiabetic, and anticancer potentials of Roselle [63]. The extracts of Roselle calyces contain a variety of anthocyanin pigments such as delphinidin-3-sambubioside, cyanidin-3-sambubioside, delphinidin-3-glucoside, cyanidin-3-*O*-glucoside, malvidin-3-*O*-glucoside, and petunidin-3-glucoside. These pigments are responsible for the red and purple colors as well as several bioactive characteristics of Roselle [7]. The TPC, TFC, and TAC values of the hydroethanolic extract of Roselle calyces were 58.83 ± 8.81 mg GAE/g, 30.44 ± 6.23 mg QUE/g, and 13.88 ± 3.66 mg cyanidine-3-*O*-glucoside equivalents of anthocyanins/g, respectively, to assess the phytochemical profile of *H. sabdariffa* calyces (Fig. 4A). In addition, the DPPH radical scavenging activity (%) and DPPH radical IC_50_ value were 98.22 ± 9.21 (%) of 150 mg Roselle/mL and 60.92 ± 6.77 mg Roselle/mL compared to 96.68 ± 6.44 (%) of 45 mg ascorbic acid/mL and 21.43 ± 3.66 mg ascorbic acid/mL, respectively, to determine the antioxidant potential of the hydroethanolic extract of *H. sabdariffa* calyces (Fig. 4B and C, Figs. S5 and S6, Tables S2 and S3). These results are consistent with the findings of a previous study [64], which demonstrated a correlation between the phytochemical screening profile and the antioxidant capacity of Roselle calyces. The hydroethanolic extract of *H. sabdariffa* calyces (60–70% *v/v* ethanolic solution) introduces the highest TFC of their calyces, which significantly reflects the antioxidant, antimicrobial, anti-inflammatory, and hypolipidemic potentials of *H. sabdariffa* flowers [16]. Moreover, the antioxidant and antibacterial activities of aqueous and alcoholic extracts of *H. sabdariffa* calyces are significantly associated with their effective phytochemical compounds [7, 32, 65, 66].

**Fig. 8:**
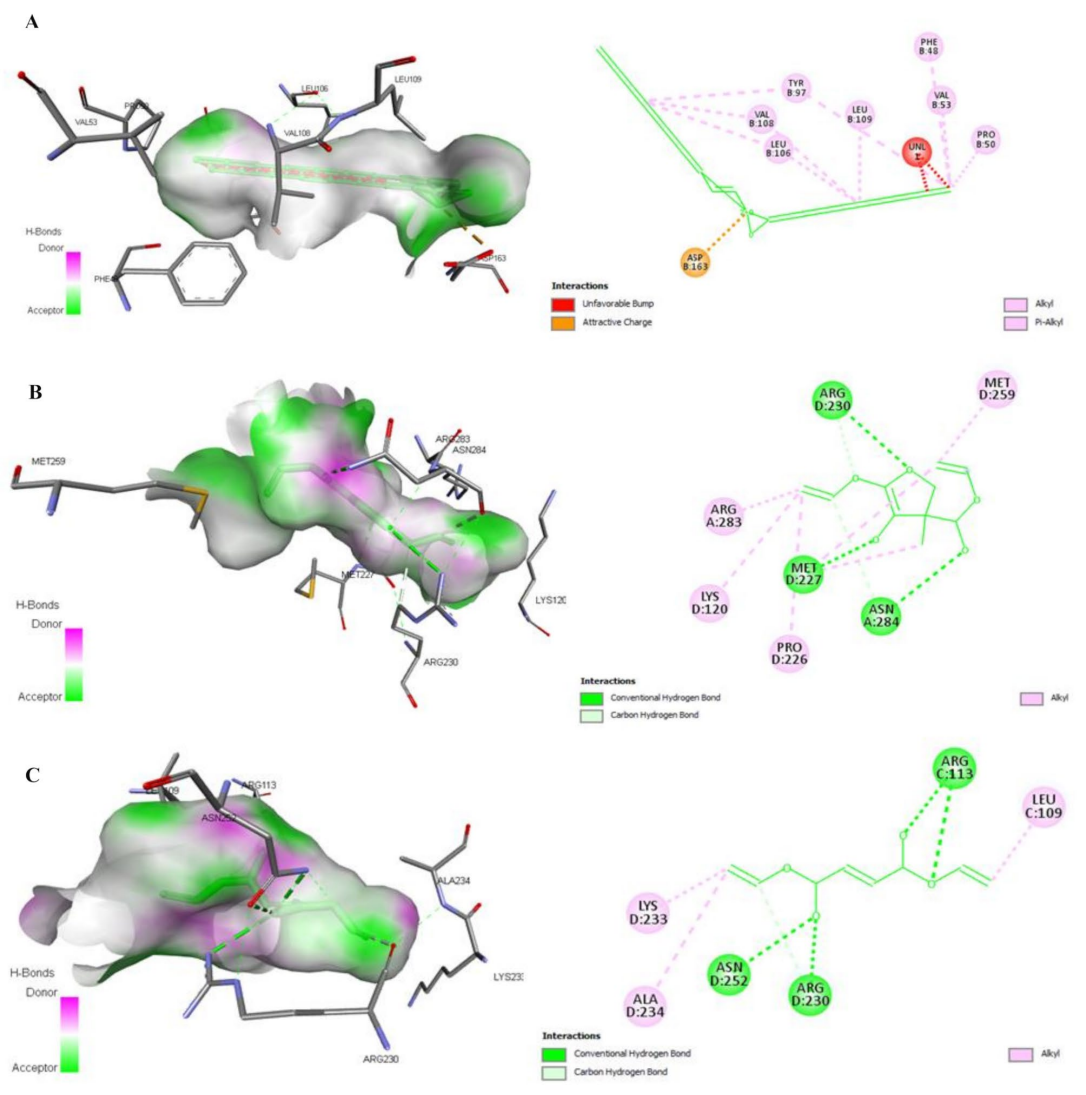
The 3D- and 2D-docked structures of the promising (*E*)-10-Octadecenoic acid methyl ester (**A**), Butanedioic acid, 3-hydroxy-2,2-dimethyl-, diethyl ester (**B**), and Diethyl succinate (**C**)-correlated *E. coli* MenB lyase complexes show active-binding site residues of MenB lyase using BIOVIA Drug Discovery Studio Visualizer software. The H-bonding (conventional, carbon, and π-Donor), electrostatic (π-Anion, π-Cation, and π-Sulfur), and hydrophobic (π-π Stacked, π-π T-Shaped, Alkyl, π-Alkyl, π-σ, and Amide-π Stacked) intermolecular interactions are demonstrated

### Antibacterial activities of the extract of *H. sabdariffa* calyces

The pathogenic bacteria significantly cause resistance against numerous traditional synthetic antibiotics and potentially develop harmful effects on the healthcare system globally. Bioactive metabolites present in a wide range of traditional medicinal plants, and they can affect the growth rate, cell survival, and pathogenicity of resistant bacteria [5, 6]. As a member of the Malvaceae family, *H. sabdariffa* L. (Roselle) is considered an edible aromatic plant that is widely used in the food industries and pharmacological applications. The bioactive phytocompounds of *H. sabdariffa* calyces are significantly associated with numerous therapeutic activities such as antimicrobial, antidiabetic, anti-obesity, antioxidant, anti-inflammatory, and anticancer [7]. Zone diameters of inhibition growth of 10 mg/mL hydroethanolic extract of *H. sabdariffa* calyces against *A. baumanii*,* E. coli*, *K. pneumoniae*, and *P. aeruginosa* were 21.66 ± 1.77 mm, 29.66 ± 2.22 mm, 29.72 ± 1.88 mm, and 24.74 ± 1.52 mm compared to 32.55 ± 1.23 mm, 32.68 ± 1.12 mm, 30.53 ± 1.38 mm, and 33.39 ± 1.41 mm of 4 mg/mL TGC, respectively (Figs. 3B-E and 4D, Figs. S4E-H, S7 and S8, Table S4). A recent study reported that the alcoholic extract of *H. sabdariffa* L. flowers effectively inhibited growth rates and reduced pathogenicity of *E. coli* (RCMB004001), *E. cloacae* (ATCC13047), *P. aeruginosa* (RCMB008001), and *S. aureus* (ATCC 25923) [3], which confirmed and matched with our findings against selective MDR clinical bacterial isolates. In this study, the hydroethanolic extract of *H. sabdariffa* calyces exhibited potent bactericidal characteristics against selective MDR clinical bacterial isolates, which aligns with the findings of previous studies [7, 67, 68]. Recent findings also demonstrated the significance of the Egyptian medicinal plant extracts as potent antibacterials against selective MDR Gram-negative and Gram-positive bacterial strains [4]. In the current study, the MIC and MBC values of the hydroethanolic extract of *H. sabdariffa* calyces against *A. baumanii*,* E. coli*, *K. pneumoniae*, and *P. aeruginosa* clinical isolates were 1.00 ± 0.123 and 1.25 ± 0.127 mg/mL, 0.50 ± 0.051 and 0.75 ± 0.034 mg/mL, 0.50 ± 0.038 and 0.75 ± 0.051 mg/mL, and 1.00 ± 0.106 and 1.25 ± 0.161 mg/mL compared to 0.04 ± 0.009 and 0.10 ± 0.022 mg/mL, 0.20 ± 0.023 and 0.20 ± 0.038 mg/mL, 0.20 ± 0.031 and 0.40 ± 0.045 mg/mL, and 0.02 ± 0.005 and 0.03 ± 0.007 mg/mL TGC, respectively (Fig. 4F and G, Table S5). Furthermore, the values of MBC/MIC ratios of the hydroethanolic extract of *H. sabdariffa* calyces against *A. baumanii*,* E. coli*, *K. pneumoniae*, and *P. aeruginosa* clinical isolates were 1.25, 1.50, 1.50, and 1.25 compared to 2.50, 1.00, 2.00, and 1.50 values of TGC, respectively (Table S5). In the present study, the bactericidal characteristics of the hydroethanolic extract of Roselle calyces against selective MDR clinical bacterial isolates are clearly related to the phytochemical characterization and the antioxidant activity of Roselle flowers. Previous research has extensively assessed the bactericidal properties of Roselle calyces [44], which matched with the results of this study. Moreover, the ClustVis heatmap distribution analysis demonstrated the effectiveness of Roselle calyces as potent antibacterials against selective MDR clinical bacterial isolates compared with the TGC administration (Fig. 4E). A sequential analysis of the OrRd distribution form was conducted. The highest values are represented by the orange color, while the lowest values are represented by the red color (http://biit.cs.ut.ee/clustvis/).

### Scanning electron microscopy (SEM) evaluation

The treated clinical isolates of *A. baumanii*, *E. coli*, *K. pneumoniae*, and *P. aeruginosa* with the hydroethanolic extract of *H. sabdariffa* calyces were examined to evaluate the changing that presented and observed in the surface morphological characteristics compared with the untreated forms. The untreated isolates of *A. baumanii*, *E. coli*, *K. pneumoniae*, and *P. aeruginosa* were regularly shown as condensed rods using SEM examination (Fig. 5A, C, E, and G). The hydroethanolic extract of *H. sabdariffa* calyces caused irregularities in the surfaces and sizes of bacterial cells, as well as division, distortion, and shrinkage of the treated bacterial cells. Additionally, the barriers between the bacterial cells were lysed. The treated forms of *A. baumanii*, *E. coli*, *K. pneumoniae*, and *P. aeruginosa* clearly exhibited these effects (Fig. 5B, D, F, and H). The findings in the SEM photomicrographs of this study were matched with previous findings that demonstrated the ultrastructural changes on the surfaces of bacterial strains after administration with selective plant extracts as effective antibacterials [69–71]. The antioxidant capacity and the antibacterial potentials of the hydroethanolic extract of Roselle calyces toward selective clinical MDR isolates were clearly observed and confirmed using SEM examination, which greatly relate to the phytochemical screening profile and the volatile aromatic components of Roselle calyces.

**Fig. 9:**
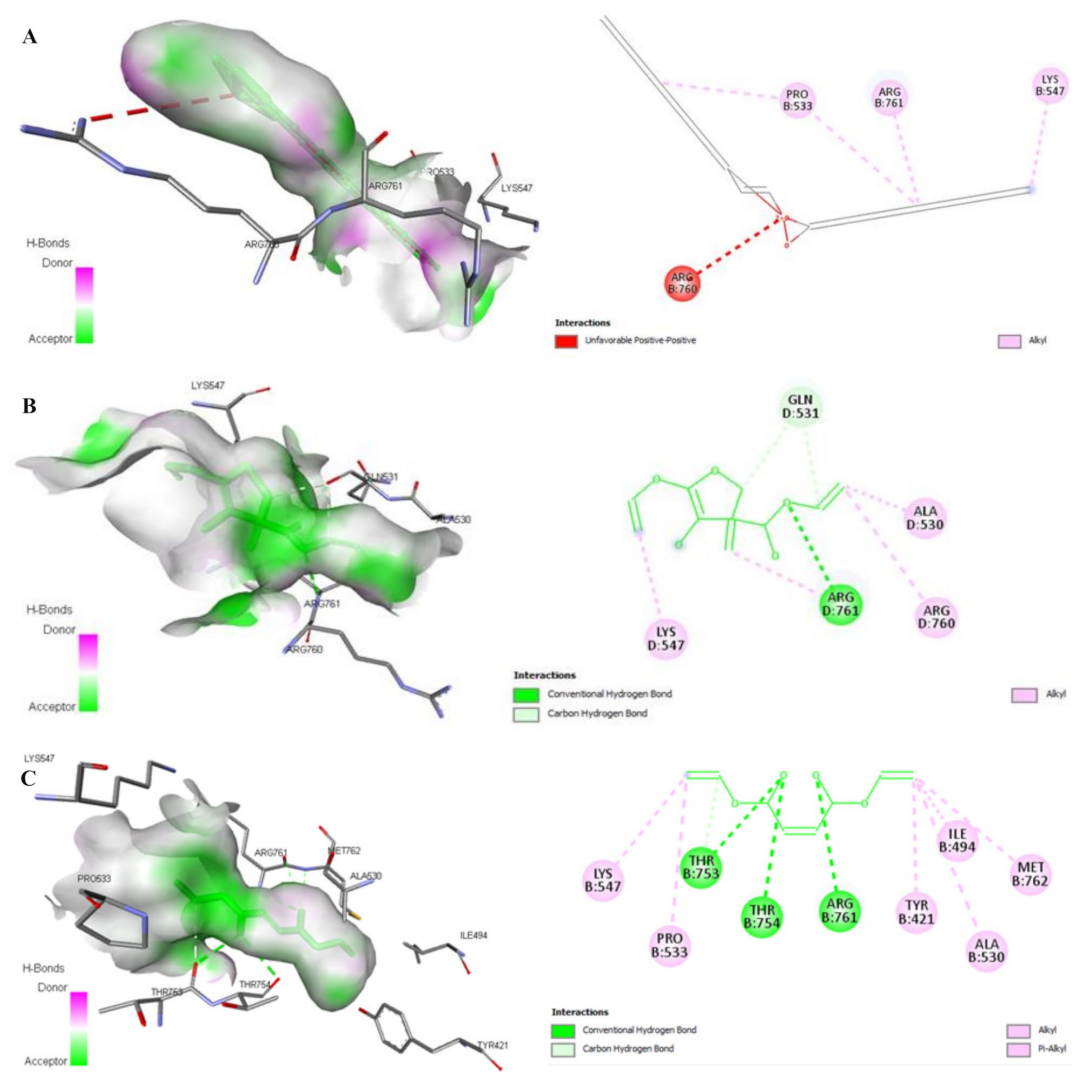
The 3D- and 2D-docked structures of the promising (*E*)-10-Octadecenoic acid methyl ester (**A**), Butanedioic acid, 3-hydroxy-2,2-dimethyl-, diethyl ester (**B**), and Diethyl succinate (**C**)-correlated *E. coli* DNA gyrase complexes show active-binding site residues of DNA gyrase using BIOVIA Drug Discovery Studio Visualizer software. The H-bonding (conventional, carbon, and π-Donor), electrostatic (π-Anion, π-Cation, and π-Sulfur), and hydrophobic (π-π Stacked, π-π T-Shaped, Alkyl, π-Alkyl, π-σ, and Amide-π Stacked) intermolecular interactions are demonstrated

### Molecular modeling simulations

The bioinformatics field correlates with computational-based tools to evaluate the biological signaling processes and provide specific, accurate predictions for selective in vitro and in vivo studies and clinical trials [72]. Molecular docking simulations determine the conformations and orientations of the putative chemicals into the active-binding pockets of their selective and promising potential targets. Searching algorithms generate conformations that are ranked according to their scoring functions [45, 46]. The antibacterial activities of the hydroethanolic extract of Roselle calyces were confirmed through constructing in silico molecular docking simulations for the main Roselle volatile compounds that identified by the GC-MS analysis toward selective antibacterial survival targets. As the most abundant volatile aromatic components of Roselle hydroethanolic extract, (*E*)-10-Octadecenoic acid methyl ester (59.23%), Butanedioic acid, 3-hydroxy-2,2-dimethyl-, diethyl ester (6.22%), and Diethyl succinate (2.35%) were simulated against selective *E. coli* targets to evaluate their antibacterial potentials. As reported in Table 3, the estimated free energy of binding and the inhibition constant (*Ki*) values of (*E*)-10-Octadecenoic acid methyl ester, Butanedioic acid, 3-hydroxy-2,2-dimethyl-, diethyl ester, and Diethyl succinate toward *E. coli* MenB lyase were − 13.14 kcal/mol and 232.39 pM, -6.59 kcal/mol and 15.53 µM, and − 6.59 kcal/mol and 15.86 µM, respectively. Furthermore, the estimated free energy of binding and the *Ki* values of (*E*)-10-Octadecenoic acid methyl ester, Butanedioic acid, 3-hydroxy-2,2-dimethyl-, diethyl ester, and Diethyl succinate toward *E. coli* DNA gyrase were − 11.54 kcal/mol and 3.45 nM, -5.59 kcal/mol and 93.87 µM, and − 5.79 kcal/mol and 65.51 µM, respectively (Table 3). The value of *Ki* represents the half-maximum inhibition of the biological functions of an enzyme by a specific inhibitor, which is used to evaluate the effectiveness of selective ligands against promising potential targets. The chemical compounds with *Ki* values ˂ 100 µM are considered potent inhibitors, whereas *Ki* values > 100 µM are non-potent inhibitors [73], that clearly reflected the potent antibacterials of Roselle volatile components in the present study (Table 3).

Figures 8 and 9 clearly demonstrate diversity of the non-covalent intermolecular interactions of (*E*)-10-Octadecenoic acid methyl ester, Butanedioic acid, 3-hydroxy-2,2-dimethyl-, diethyl ester, and Diethyl succinate with active amino acid residues of their selective promising antibacterial *E. coli* MenB lyase and DNA gyrase targets as H-bonding (conventional, carbon, and π-Donor), electrostatic (π-Cation, π-Anion, π-Sulfur, and attractive charges), and hydrophobic (π-π Stacked, π-π T-Shaped, Alkyl, π-Alkyl, π-σ, and Amide-π Stacked) interactions. According to the scoring functions, the strength of the binding modes is significantly associated with the intermolecular interactions between the chemical compounds and their selective promising targets as classical and/or non-classical H-bonds, electrostatic, and hydrophobic interactions [46], which match with our in silico findings. Based on the findings presented in Figs. 6-9; Table 4, the main volatile components of *H. sabdariffa* calyces demonstrated a correlation with antibacterial activity against *E. coli* MenB lyase or DNA gyrase targets. The intermolecular interactions of (*E*)-10-Octadecenoic acid methyl ester, Butanedioic acid, 3-hydroxy-2,2-dimethyl-, diethyl ester, and Diethyl succinate with the key amino acid residues of *E. coli* MenB lyase or DNA gyrase targets exhibited different types, strength, and bond lengths. Moreover, the (*E*)-10-Octadecenoic acid methyl ester, Butanedioic acid, 3-hydroxy-2,2-dimethyl-, diethyl ester, and Diethyl succinate-interacted complexes potentially demonstrated diversity of the conventional and carbon H-bonding, electrostatic, and hydrophobic intermolecular interactions toward their selective promising antibacterial *E. coli* MenB lyase and DNA gyrase (Figs. 6-9; Table 4). The properties of H-bonding intermolecular interactions as H-bond interaction order, HBD, HBA, and H-bond distance (Å) are depicted in Table 4. The H-bonding potentially enhances the binding affinities of ligands toward their potential targets. This plays a significant role in the development of drugs that target specific chemical and biological processes, molecular recognition, and biological activities [74]. 

This study examined the scoring functions, binding affinities, and non-covalent intermolecular interactions of three main active compounds of Roselle: (*E*)-10-Octadecenoic acid methyl ester (59.23%), Butanedioic acid, 3-hydroxy-2,2-dimethyl-, diethyl ester (6.22%), and Diethyl succinate (2.35%). These interactions were analyzed in relation to their antibacterial activity against *E. coli* MenB lyase or DNA gyrase. Figures 6-9; Tables 3 and 4 demonstrate the strength of the binding modes, stability of the binding conformations, and diversity of the intermolecular interactions that presented in the *E. coli* MenB lyase or DNA gyrase complexes. These findings align with those reported by a recent study [75]. The number of H-bonds is markedly correlated with the binding mode efficiency and the inhibition potentials [73] that matched with the antibacterial potentials of the main volatile components of the hydroethanolic extract of *H. sabdariffa* calyces against selective MDR clinical bacterial isolates. 

The presence of hydroxyl (OH) groups in fatty acid derivatives significantly disrupts the structural integrity and uniformity of bacterial cell membranes [76]. Fatty acids possess amphipathic properties that greatly enhance their capacity to dissolve various components of bacterial cell membranes, such as proteins and lipid bilayers. This process leads to the development of cell lysis characteristics [77]. Menaquinone, a polyisoprenylated naphthoquinone, is considered a redox-active cofactor that greatly upregulates the electron transport respiratory chain to generate energy (ATP) for most Gram-positive and some Gram-negative bacteria under anaerobic conditions [78]. For drug design development, the structure-based (SB)-mediated antibacterial drugs selectively target enzymes that regulate the menaquinone (vitamin K_2_) biosynthesis pathway, such as 1,4-dihydroxy-2-naphthoyl-CoA synthase (MenB) [79]. The *E. coli* MenB crotonase enzyme facilitates the production of the second aromatic ring of naphthoquinone (1,4-dihydroxy-2-naphthoyl-CoA (DHNA-CoA)) from *O*-succinylbenzoyl-CoA (OSB-CoA) through a process called anaerobic respiration. This process involves the succinyl side chain of *O*-succinylbenzoate (OSB) and utilizes intramolecular Claisen or Dieckmann condensation processes in the menaquinone biosynthetsis pathway [80]. The main volatile aromatic components of the hydroethanolic extract of *H. sabdariffa* calyces in this study were (*E*)-10-Octadecenoic acid methyl ester (59.23%), Butanedioic acid, 3-hydroxy-2,2-dimethyl-, diethyl ester (6.22%), and Diethyl succinate (2.35%). These components were identified as potent *E. coli* MenB competitive inhibitors (OSB-CoA analogs or DHNA-CoA synthase inhibitors), which have the potentials to disrupt *E. coli* DHNA-CoA and menaquinone biosynthesis, anaerobic respiration process, energy generation, bacterial growth rate, and cell survival. The results of our study were validated and found to be consistent with other molecular simulations [3, 80]. Furthermore, the side chains of Asp163, Val108, Tyr97, Leu106, Leu109, Phe48, Asn284, Arg283, and Met259, as well as other key amino acid residues, were observed in the catalytic active-binding cavity of *E. coli* MenB lyase with (*E*)-10-Octadecenoic acid methyl ester, Butanedioic acid, 3-hydroxy-2,2-dimethyl-, diethyl ester, and Diethyl succinate as *E. coli* MenB lyase competitive inhibitors/OSB-CoA analogs (Fig. 8; Table 4). These results align with those of a previous study [47]. These amino acid residues clearly present in the catalytic active-binding pocket of *E. coli* MenB lyase that responsible for the recognition and the binding of the OSB-CoA substrate to upregulate the activity of *E. coli* MenB lyase and to induce the cell survival and the pathogenicity of *E. coli* [81, 82].

DNA gyrase, a type IIA DNA topoisomerase (Top2), is an essential enzyme that controls the cellular homeostasis of DNA supercoiling. It is effectively targeted by specific inhibitors of bacterial topoisomerases. The promising drug-mediated DNA gyrase nucleoprotein complexes potentially target the DNA cleavage activity of *E. coli* DNA gyrase [48]. The *E. coli* DNA gyrase GyrB subunit (B and D chains) includes an insertion domain that facilitates communication between the various functional domains of the GyrB subunit. The deletion or disruption of this domain significantly impairs the ability of *E. coli* DNA gyrase to attach to DNA and reduces its ATP hydrolysis and DNA-negative supercoiling activities [83]. In the present study, the main volatile aromatic components of the hydroethanolic extract of *H. sabdariffa* calyces included (*E*)-10-Octadecenoic acid methyl ester (59.23%), Butanedioic acid, 3-hydroxy-2,2-dimethyl-, diethyl ester (6.22%), and Diethyl succinate (2.35%). These compounds can potentially disrupt the enzymatic activity of the GyrB subunit of *E. coli* DNA gyrase. These volatile components form non-covalent intermolecular interactions with the active amino acid residues (Tyr421B, Ile494B, Pro533B, Ala530B, Lys547B, Gln531D, Lys547D, Ala530D, Arg760B, Arg761B, Arg761D, Arg760D, Met762B, Thr753B, and Thr754B) that present in the GyrB TOPRIM insertion domains of the *E. coli* DNA gyrase B and D chains (Fig. 9; Table 4). As the most abundant volatile aromatic components of the hydroethanolic extract of *H. sabdariffa* calyces, (*E*)-10-Octadecenoic acid methyl ester (59.23%), Butanedioic acid, 3-hydroxy-2,2-dimethyl-, diethyl ester (6.22%), and Diethyl succinate (2.35%) could impair the ability of *E. coli* DNA gyrase to attach to DNA, reduce its ATP hydrolysis and DNA-negative supercoiling activities, and inhibit the cell survival and the pathogenicity of *E. coli*. These findings confirmed importance of the GyrB TOPRIM insertion domains in the DNA cleavage activity of the *E. coli* DNA gyrase GyrB subunit [48].

**Table 1:** The GC-MS analytical report of the hydroethanolic extract of Roselle calyces

Peak number	Retention time (RT)*	Peak area%#	SI^A^	RSI^B^	Probability%	Volatile organic compounds^C^	MF & MW
1	7.56	1.38	515	537	10.81	Glucose diethylmercaptal	C_10_H_22_O_5_S_2_; 286
2	9.14	1.33	822	822	92.72	1,1-Diethoxypropane	C_7_H_16_O_2_; 132
3	10.95	0.72	805	825	15.93	*O*-Benzylhydroxylamine hydrochloride	C_7_H_10_ClNO; 123
4	11.20	0.95	743	802	12.58	1-Propanone, 1-phenyl-3-[2-(phenylmethoxy)phenyl]-	C_22_H_20_O_2_; 316
5	12.04	0.44	797	800	21.94	*P*-Xylene (1,4-Dimethylbenzene)	C_8_H_10_; 106
6	20.93	0.67	852	852	95.09	Diethyl succinate/1,4-Diethyl butanedioate/Butanedioic acid, diethyl ester	C_8_H_14_O_4_; 174
7	22.14	1.05	507	512	42.08	1,4-Dimethyl-3-n-octadecylcyclohexane	C_26_H_52_; 364
8	23.09	1.10	905	907	95.77	Diethyl malate/Diethyl 2-hydroxysuccinate/diethyl 2-hydroxybutanedioate/Malic acid, diethyl ester/Butanedioic acid, hydroxy-, diethyl ester, (ñ)-	C_8_H_14_O_5_; 190
9	23.72	1.18	591	666	24.03	Propionic acid, 3-(*p-*cyanobenzoyl)-2-methyl-	C_12_H_11_NO_3_; 217
10	23.82	0.37	769	796	59.41	5-Hydroxymethylfurfural/5-Hydroxymethyl-2-furaldehyde/5-(hydroxymethyl)furan-2-carbaldehyde/2-Furancarboxaldehyde, 5-(hydroxymethyl)-	C_6_H_6_O_3_; 126
11	24.70	1.68	783	793	86.55	Diethyl succinate/1,4-Diethyl butanedioate/Butanedioic acid, diethyl ester	C_8_H_14_O_4_; 174
12	25.20	59.23	811	811	9.13	(*E*)-10-Octadecenoic acid methyl ester/ Methyl trans-10-octadecenoate/ methyl (*E*)-octadec-10-enoate	C_19_H_36_O_2_; 296
13	25.58	3.06	742	755	8.21	8,11-Octadecadienoic acid, methyl ester	C_19_H_34_O_2_; 294
14	25.97	0.32	662	756	11.75	Dodecanoic acid, methyl ester/Methyl dodecanoate/Methyl laurate/Lauric acid methyl ester	C_13_H_26_O_2_; 214
15	26.44	2.31	673	720	29.95	Heptadecanoic acid, 16-methyl-, methyl ester/16-Methylheptadecanoic acid methyl ester/Methyl isostearate/Methyl 16-methylheptadecanoate	C_19_H_38_O_2_; 298
16	27.14	1.08	421	429	19.62	Prost-13-en-1-oic acid, 9-(methoxyimino)-11,15-*bis*[(trimethylsilyl)oxy]-, trimethylsilyl ester, (8.xi.,12.xi.)-/Trimethylsilyl 9-(methoxyimino)-11,15-*bis*[(trimethylsilyl)oxy]prost-13-en-1-oate	C_30_H_61_NO_5_Si_3_; 599
17	27.21	0.68	425	447	3.45	Oleic acid/*cis*-9-Octadecenoic acid/Elaidoic acid/(*Z*)-octadec-9-enoic acid	C_18_H_34_O_2_; 282
18	27.32	0.84	493	510	6.35	1,18-Octadecanedioic acid/1,16-Hexadecanedicarboxylic acid/Octadecane-1,18-dioic acid/1,18-Octadecadioic acid	C_18_H_34_O_4_; 314

**Table 2:** The GC-MS analytical report of the hydroethanolic extract of Roselle calyces (*continued*)

Peak number	Retention time (RT)*	Peak area%#	SI^A^	RSI^B^	Probability%	Volatile organic compounds^C^	MF & MW
19	27.89	0.71	510	702	55.96	*N-*(2-Hydroxyethyl)decanamide/capric monoethanolamide/capryl ethanolamide/*N*-decanoylethanolamine	C_12_H_25_NO_2_; 215
20	28.00	0.30	493	629	6.65	4,4’-Biscyclohexanone, 2,2’,6,6’-tetramethyl-/4-(3,5-dimethyl-4-oxocyclohexyl)-2,6-dimethylcyclohexan-1-one	C_16_H_26_O_2_; 250
21	28.71	4.27	807	827	17.72	8,11-Octadecadienoic acid, methyl ester	C_19_H_34_O_2_; 294
22	29.03	0.71	510	515	5.65	1,3,5-trimethyl-2-octadecylcyclohexane/1,5-Trimethyl-4-n-octadecylcyclohexane	C_27_H_54_; 378
23	29.28	4.17	656	680	11.56	8,11-Octadecadienoic acid, methyl ester	C_19_H_34_O_2_; 294
24	30.75	1.20	614	644	7.14	Cyclopropaneoctanoic acid, 2-[(2-pentylcyclopropyl)methyl]-, methyl ester/ Methyl 8-[2-[(2-pentylcyclopropyl)methyl]cyclopropyl]octanoate	C_21_H_38_O_2_; 322
25	31.56	0.95	485	548	7.32	α-*D*-Glucopyranoside, methyl 2-(acetylamino)-2-deoxy-3-*O*-(trimethylsilyl)-, cyclic butylboronate	C_16_H_32_BNO_6_Si; 374
26	31.96	6.22	673	693	80.17	Butanedioic acid, 3-hydroxy-2,2-dimethyl-, diethyl ester/diethyl 3-hydroxy-2,2-dimethylbutanedioate	C_10_H_18_O_5_; 218
27	32.21	1.21	554	689	36.86	2,6-Piperidinedione, 3-phenyl-/ 3-phenylpiperidine-2,6-dione/ 2-Phenylglutarimide/ Glutarimide, 2-phenyl-	C_11_H_11_NO_2_; 189
28	34.52	0.73	696	716	35.27	Pentadecanoic acid, 14-methyl-, methyl ester/Methyl 14-methylpentadecanoate/14-Methylpentadecanoic acid methyl ester/Methyl isohexadecanoate	C_17_H_34_O_2_; 270
29	36.87	0.52	347	350	10.74	Hexadecanoic acid, 1a,2,5,5a,6,9,10,10a-octahydro-5,5a-dihydroxy-4-(h ydroxymethyl)-1,1,7,9-tetramethyl-11-oxo-*1 H*-2,8a-methanocyclopenta[a]cyclopropa[e]cyclodecen-6-yl ester, [1a*R*-(1aà,2à,5á,5aá,6á,8aà,9à,10aà)]-	C_36_H_58_O_6_; 586
30	37.03	0.64	444	500	4.41	Hexadecanoic acid, ethyl ester/Ethyl palmitate/Ethyl hexadecanoate/Palmitic acid ethyl ester	C_18_H_36_O_2_; 284

*, Min; **#**, relative concentration% of Roselle volatile aromatic components; **A**, calculated retention indices related to C_7_ - C_36_ n-alkanes on the chromatography DB-5 capillary column; **B**, reference standard retention indices (reported/literature retention indices (LRIs)); **C**, identification of the volatile aromatic components by comparing their retention indices (RI) as GC characteristics and/or comparing their mass spectra as MS characteristics with LRIs and mass spectra of literature data (NIST and WILEY libraries database); **MF & MW**, molecular formula and molecular weights (g/mol) of twenty-seven Roselle volatile phytochemicals

**Table 3:** The binding scores (kcal/mol), binding affinities (*Ki*), and the stabilities (RMSD-tolerance) of selective promising *H. sabdariffa* calyx volatile components-correlated antibacterial *E. coli* MenB lyase or DNA gyrase complexes

Antibacterial-correlated leads	Binding scoring	Ki	RMSD-Tolerance (rmstol) of 2.00Å
	E. coli MenB lyase		
(*E*)-10-Octadecenoic acid methyl ester	-13.14	232.39 pM	0.65
Butanedioic acid, 3-hydroxy-2,2-dimethyl-, diethyl ester	-6.59	15.53 µM	0.88
Diethyl succinate	-6.59	15.86 µM	0.76
	*E. coli* DNA gyrase (isomerase)		
(*E*)-10-Octadecenoic acid methyl ester	-11.54	3.45 nM	0.65
Butanedioic acid, 3-hydroxy-2,2-dimethyl-, diethyl ester	-5.59	93.87 µM	0.94
Diethyl succinate	-5.79	65.51 µM	0.94

Cα-RMSD-tol, root-mean-square deviation values reflect the stability of the protein-ligand complex conformations

**Table 4:** Properties of the H-bond interactions of selective promising *H. sabdariffa* calyx volatile components-correlated antibacterial *E. Coli* MenB lyase or DNA gyrase complexes

Selective antibacterial leads	H-bond order	HBD	HBA	H-bond distance (Å)
		E. coli MenB lyase		
Butanedioic acid, 3-hydroxy-2,2-dimethyl-, diethyl ester	**3** Conventional H-bond **2** Carbon H-bond	A: ASN284:ND2 D: MET227:N D: ARG230:NH1 D: ARG230:CD Ligand (C)	Ligand (O) Ligand (O) Ligand (O) Ligand (O) A: ASN284:O	2.85 3.26 2.98 3.02 3.29
Diethyl succinate	**5** Conventional H-bond **1** Carbon H-bond	C: ARG113:NH1 C: ARG113:NH2 D: ARG230:NH2 D: ASN252:ND2 Ligand (O) Ligand (C)	Ligand (O) Ligand (O) Ligand (O) Ligand (O) D: ASN252:OD1 D: ARG230:O	3.03 3.08 2.73 2.95 3.17 2.96
*E. coli* DNA gyrase (isomerase)				
Butanedioic acid, 3-hydroxy-2,2-dimethyl-, diethyl ester	**1** Conventional H-bond **2** Carbon H-bond	D: ARG761:N Ligand (C) Ligand (C)	Ligand (O) D: GLN531:O D: GLN531:O	2.89 2.73 2.96
Diethyl succinate	**3** Conventional H-bond **1** Carbon H-bond	B: ARG761:N Ligand (O) Ligand (O) Ligand (C)	Ligand (O) B: THR753:O B: THR754:O B: THR753:O	3.29 2.69 3.22 3.19

Active key amino acid residues (active catalytic-binding pocket) were reported. HBD represents the H-bond donor, while HBA represents the H-bond acceptor

## Conclusions

Based on the in vitro and in silico findings, the hydroethanolic extract of *H. sabdariffa* calyces contains polyphenols, flavonoids, and anthocyanins. It also possesses volatile aromatic components that exhibit antioxidant and antibacterial properties. These components have a broad impact on selective MDR Gram-negative clinical bacterial isolates, reducing their pathogenicity.

### Future implications

These in vitro and in silico findings confirmed the antibacterial potentials of the main volatile organic ingredients of Roselle as effective promising *E. coli* MenB lyase and DNA gyrase inhibitors. The results of this study identified survival targets to develop potentials of the antibacterial drugs. In future, we will focuse on using the classical biochemical and transcriptomic approaches to identify the promising antibacterial survival targets. Furthermore, the gene expression of these targets will be determined after treatment with the main volatile aromatic ingredients of the hydroethanolic extract of *H. sabdariffa* calyces as effective promising inhibitors against selective MDR bacterial isolates.

## Electronic supplementary material

Below is the link to the electronic supplementary material.

Supplementary Material 1

## Data Availability

‘’The authors declare that the data supporting the findings of this study are available within the main manuscript and additional information files. The datasets generated and/or analyzed during the current study are available from the corresponding author upon reasonable request. Source data are provided within this research article’’.

## Abbreviations

AlCl_3_: Aluminum chloride
DHNA-CoA: 1,4-dihydroxy-2-naphthoyl-CoA
DMSO: Dimethyl sulfoxide
DPPH: 1,1-diphenyl-2-picrylhydrazyl
GC-MS: Gas chromatography-mass spectrometry
GN: Gram-negative
MDR: Multidrug-resistant
MenB: 1,4-dihydroxy-2-naphthoyl-CoA synthase
NaCO_3_: Sodium carbonate
OSB-CoA: *O*-succinylbenzoyl-CoA
SEM: Scanning electron microscopy
TAC: Total anthocyanin content
TFC: Total flavonoid content
TGC: Tigecycline
TPC: Total phenolic content
XDR: Extensive drug-resistant
