# Supplementary material for: Phytochemical and antibacterial properties of calyces Hibiscus sabdariffa L.: an in vitro and in silico multitarget-mediated antibacterial study
Source: BMC Complement Med Ther. 2025 Feb 18;25:62. doi: 10.1186/s12906-025-04794-1 (PMC11837655; doi:10.1186/s12906-025-04794-1)
Supplement: Supplementary file 1 — Supplementary Material 1 [file 12906_2025_4794_MOESM1_ESM.docx]

# Phytochemical and antibacterial properties of calyces *Hibiscus sabdariffa* L.: an *in vitro* and *in silico* multitarget-mediated antibacterial study

Hend Khairy Fekry Ghaly^1^, Fatema Aly Al-Yamany Younis^2,3,*^, Azza Mahmoud Soliman^1^, and Sabha Mahmoud El-Sabbagh^1,*^

**^1^** Botany and Microbiology Department, Faculty of Science, Menoufia University, Menoufia, Shebin El-Kom, Egypt; [Hend.khairy@science.menofia.edu.eg](mailto:Hend.khairy@science.menofia.edu.eg), [azzamahmoud@science.menofia.edu.eg](mailto:azzamahmoud@science.menofia.edu.eg), and [Sabha@science.menofia.edu.eg](mailto:Sabha@science.menofia.edu.eg).

**^2^** Chemistry Department, Faculty of Science, Al-Azhar University (Girls Branch), Egypt; [fatema_aly@azhar.edu.eg](mailto:fatema_aly@azhar.edu.eg).

**^3^** Biochemistry Department, Faculty of Science, Alexandria University, Alexandria 21515, Egypt**;** [fatma.ali_PG@alexu.edu.eg](mailto:fatma.ali_PG@alexu.edu.eg) and [fatmaali201782@gmail.com](mailto:fatmaali201782@gmail.com); **ORCID ID:** 0009-0008-8759-0723; **Tel.**: +0201284064959 and +0201017467950.

***** Corresponding author: [fatema_aly@azhar.edu.eg](mailto:fatema_aly@azhar.edu.eg) and [fatma.ali_PG@alexu.edu.eg](mailto:fatma.ali_PG@alexu.edu.eg); **ORCID ID:** 0009-0008-8759-0723; **Tel.**: +0201284064959 and +0201017467950.

***** Corresponding author: [Sabha@science.menofia.edu.eg](mailto:Sabha@science.menofia.edu.eg).

# Results

| 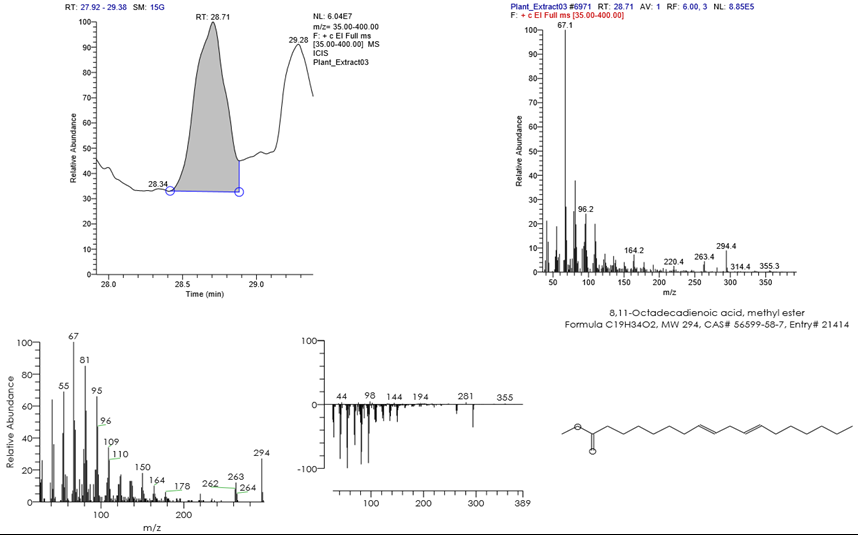  **A** |
| --- |
| 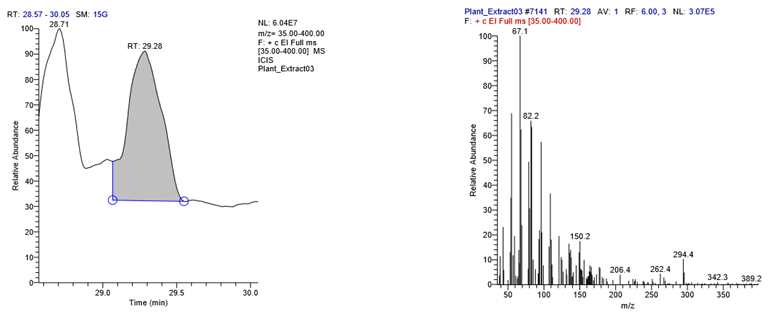  **B** |
| 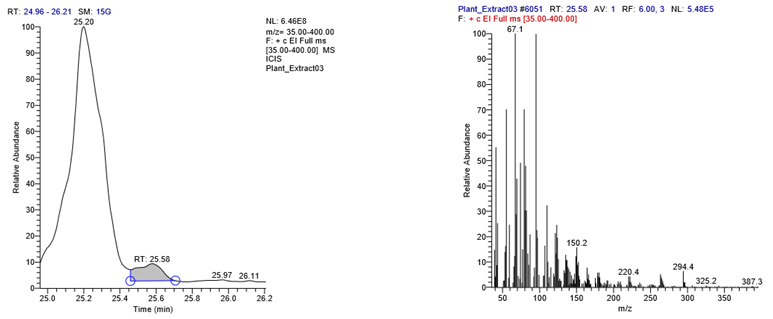  **C** |

Fig. S1 The GC-MS analysis plot, mass spectrum, and 2D-molecular structure of 8,11-Octadecadienoic acid, methyl ester as (A) 28.71 RT and 4.27%, (B) 29.28 RT and 4.17%, and (C) 25.58 RT and 3.06% fractionations

| 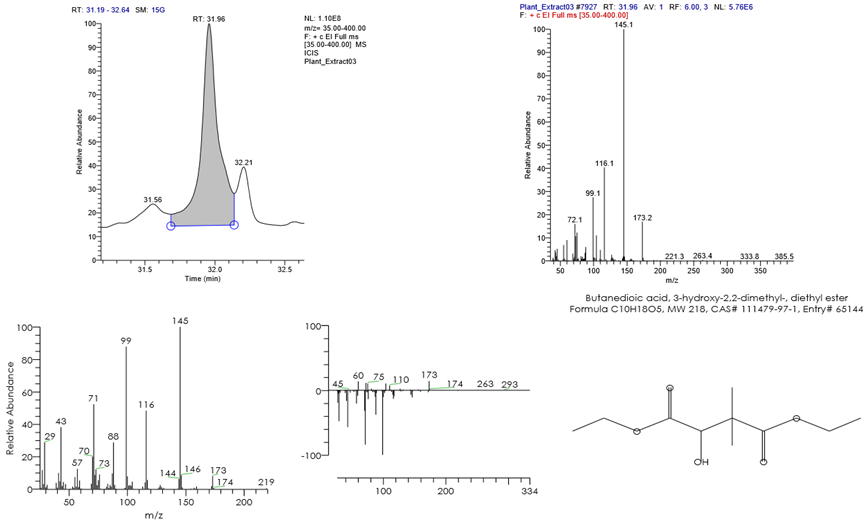  **A** |
| --- |
| 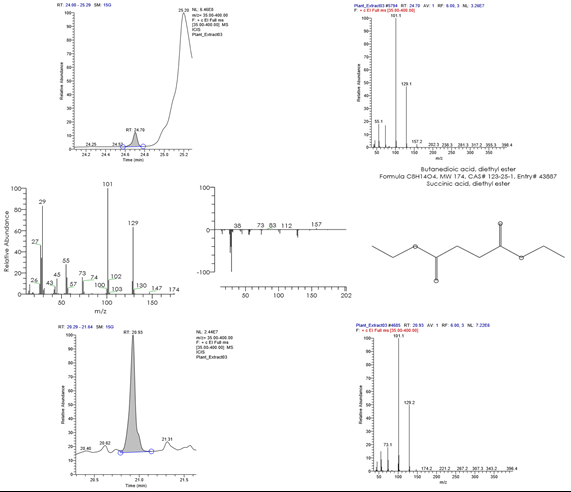  **B** |

Fig. S2 The GC-MS analysis plot, mass spectrum, and 2D-molecular structures of Butanedioic acid, 3-hydroxy-2,2-dimethyl-, diethyl ester as (A) 31.96 RT and 6.22% and Diethyl succinate/Butanedioic acid, diethyl ester as (B) 24.70 RT and 1.68% as well as 20.93 RT and 0.67% fractionations, respectively


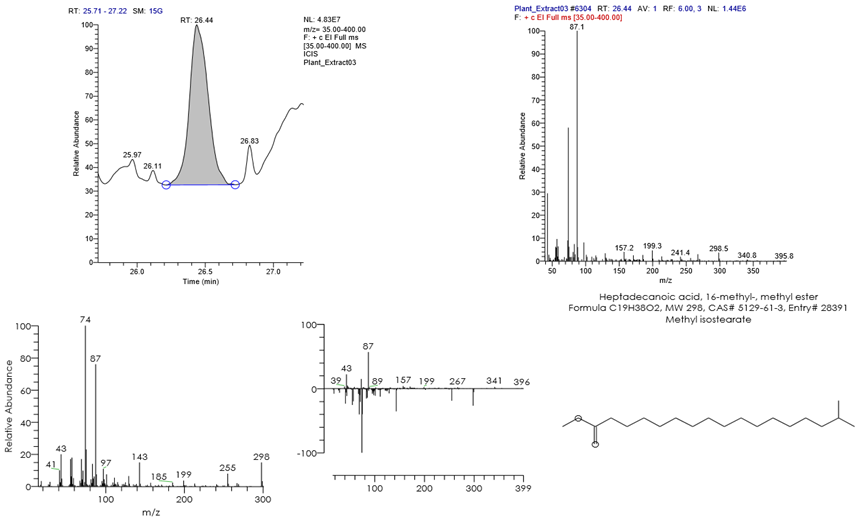


Fig. S3 The GC-MS analysis plot, mass spectrum, and 2D-molecular structure of Heptadecanoic acid, 16-methyl-, methyl ester/Methyl isostearate as 26.44 RT and 2.31% fractionation

Table S1 The antimicrobial susceptibility testing (AST) toward selective clinical bacterial isolates

| Standard broad-spectrum antibiotics | Antibiotic concentration (μg/disc) | *A. baumanii* | *E. coli* | *K. pneumoniae* | *P. aeruginosa* |
| --- | --- | --- | --- | --- | --- |
| Amikacin (AK) | 30 | Resistant | Resistant | Resistant | Resistant |
| Amoxicillin-clavulonic acid (AMC) | 30 (20/10) | Resistant | Resistant | Resistant | Resistant |
| Ampicillin-sulbactum (SAM) | 20 | Resistant | Resistant | Resistant | Resistant |
| Cefoprazone-sulbectam (CFS) | 15/30 | Resistant | Resistant | Resistant | **Weak susceptible** |
| Cefotaxime (CTX) | 30 | Resistant | Resistant | Resistant | Resistant |
| Ceftazidime (CAZ) | 30 | Resistant | Resistant | Resistant | Resistant |
| Ceftazidime-avibactam (CZA) | 30 | **Moderate susceptible** | Resistant | **Weak susceptible** | Resistant |
| Cefuroxime (CXM) | 30 | Resistant | Resistant | Resistant | Resistant |
| Ciproﬂoxacin (CIP) | 5 | Resistant | Resistant | Resistant | Resistant |
| Doxycycline (DO) | 30 | **Weak susceptible** | **Moderate susceptible** | **Weak susceptible** | Resistant |
| Erythromycin (E/ERY) | 15 | Resistant | Resistant | Resistant | Resistant |
| Gentamicin (GEN/CN) | 10 | Resistant | Resistant | Resistant | Resistant |
| Imipenem (IPM) | 10 | Resistant | Resistant | Resistant | Resistant |
| Meropenem (MEM) | 10 | Resistant | Resistant | Resistant | Resistant |
| Norfloxacin (NOR) | 10 | Resistant | Resistant | Resistant | Resistant |
| Ofloxacin (OFX) | 5 | Resistant | Resistant | Resistant | Resistant |
| Piperacillin-tazobactam (TZP) | 110 | Resistant | Resistant | Resistant | Resistant |
| Sulphamethazone-trimethoprim (SXT) | 25 (23.75/1.25) | Resistant | Resistant | Resistant | Resistant |
| Tetracycline (TE) | 30 | Resistant | Resistant | Resistant | Resistant |
| Tigecycline (TGC) | 10 | **Moderate susceptible** | **Moderate susceptible** | **Moderate susceptible** | **Moderate susceptible** |
| Vancomycin (VA) | 30 | Resistant | Resistant | Resistant | Resistant |

| 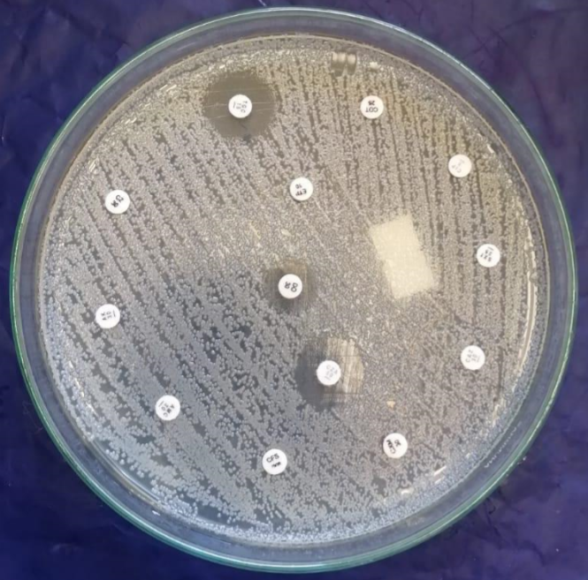  **A** | 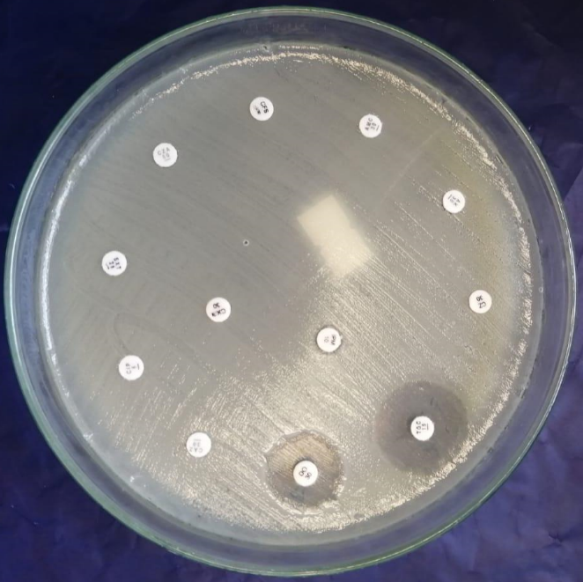  **B** | 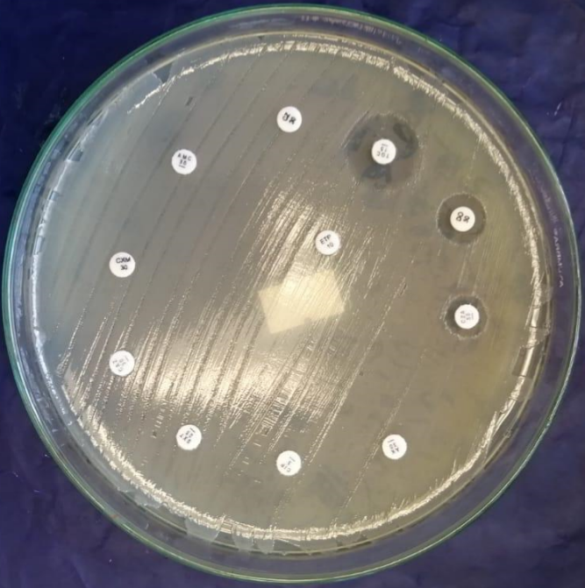  **C** | 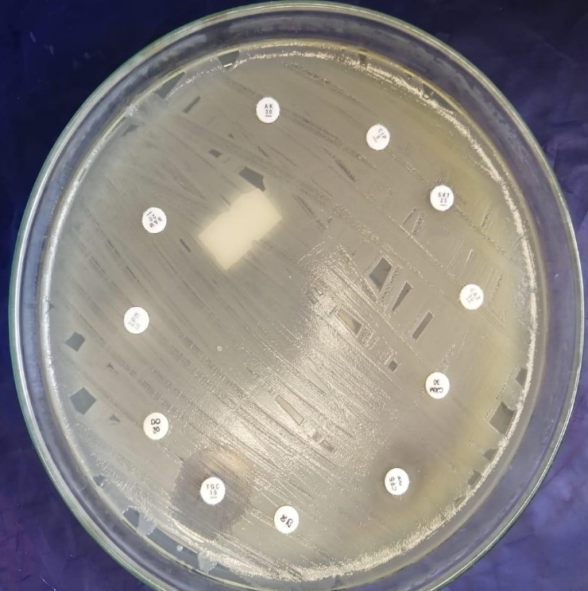  **D** |
| --- | --- | --- | --- |
| 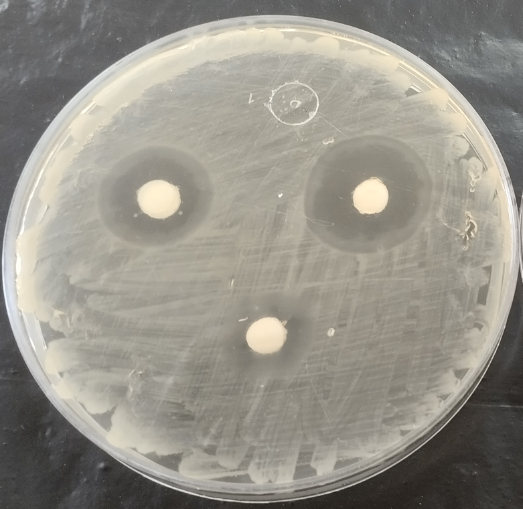  **5 μg**  **1 μg**  **15 μg**  **E** | 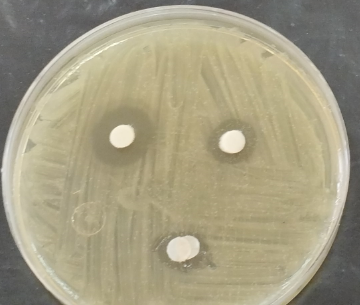  **1 μg**  **5 μg**  **15 μg**  **F** | 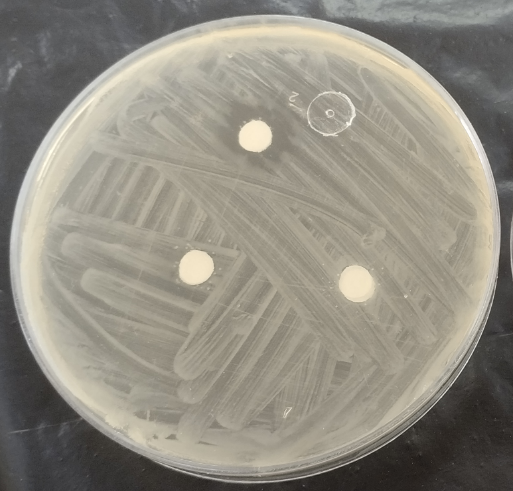  **15 μg**  **5 μg**  **1 μg**  **G** | 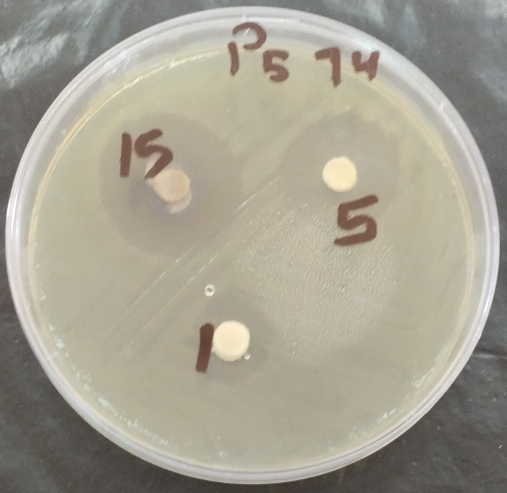  **H** |

Fig. S4 The antimicrobial susceptibility testing (AST) and the zone diameters of inhibition growth (mm) of TGC against selective clinical MDR bacterial isolates such as *A. baumanii* (A and E)*, E. coli* (B and F), *K. pneumoniae* (C and G), and *P. aeruginosa* (D and H), respectively. Zone diameters of inhibition growth (mm) of 0.6 (15 μg/disc), 0.2 (5 μg/disc), and 0.04 (1 μg/disc) mg/ mL TGC were clearly demonstrated (E-H)

| **A** |
| --- |
| **B** |

Fig. S5 The DPPH radical scavenging activities (%) of the hydroethanolic extract of Roselle calyces at different concentrations (mg/mL) (A) compared to ascorbic acid as a reference standard antioxidant compound (B)

| **A**  **B** |
| --- |
|  |

Fig. S6 The linear regression equation and the DPPH radical scavenging activities (%) of the hydroethanolic extract of Roselle calyces at different concentrations (mg/mL) (A) compared with ascorbic acid as a reference standard antioxidant compound (B)

Table S2 The DPPH radical scavenging activities (%) of the hydroethanolic extract of Roselle calyces at different concentrations (mg/mL) and its DPPH IC_50_ value

| Roselle concentration (mg/mL) | DPPH radical scavenging activity (%) |
| --- | --- |
| 1 | 2.88 ± 0.51 |
| 5 | 5.55 ± 0.82 |
| 10 | 12.44 ± 1.55 |
| 20 | 25.66 ± 3.44 |
| 40 | 45.44 ± 4.77 |
| 60 | 62.66 ± 7.11 |
| 80 | 77.39 ± 6.88 |
| 100 | 89.77 ± 7.52 |
| 150 | 98.22 ± 9.21 |
| 200 | 112.77 ± 12.55 |
| DPPH IC_50_ value | 60.92 ± 6.77 mg Roselle/mL |

Table S3 The DPPH radical scavenging activities (%) of ascorbic acid as a reference standard antioxidant compound at different concentrations (mg/mL) and its DPPH IC_50_ value

| Ascorbic acid concentration (mg/mL) | DPPH radical scavenging activity (%) |
| --- | --- |
| 1 | 5.44 ± 0.41 |
| 5 | 12.88 ± 1.45 |
| 10 | 26.77 ± 4.88 |
| 15 | 36.66 ± 5.62 |
| 20 | 49.77 ± 7.44 |
| 25 | 57.44 ± 6.33 |
| 30 | 69.66 ± 5.72 |
| 35 | 78.77 ± 8.22 |
| 40 | 90.88 ± 5.66 |
| 45 | 96.68 ± 6.44 |
| DPPH IC_50_ value | 21.43 ± 3.66 mg ascorbic acid/ mL |

|  |  |
| --- | --- |
|  |  |

Fig. S7 The antibacterial potentials of the hydroethanolic extract of *H. sabdariﬀa* calyces against selective clinical MDR bacterial isolates

|  |  |
| --- | --- |
|  |  |

Fig. S8 The antibacterial potentials of TGC standard antibiotic against selective clinical MDR bacterial isolates

**Table S4** The zone diameters of inhibition growth (mm) of the hydroethanolic extract of dried Roselle calyces against selective MDR bacterial clinical isolates compared with TGC as a reference standard antibiotic

| MDR clinical isolates | Roselle concentration (mg/mL) | | | | | | | TGC concentration (mg/mL) | | | | | | |
| --- | --- | --- | --- | --- | --- | --- | --- | --- | --- | --- | --- | --- | --- | --- |
|  | 10 | 6 | 4 | 2 | 1 | 0.5 | 0.1 | 4 | 2 | 1.2 | 0.6 | 0.2 | 0.04 | 0.02 |
| *A. baumanii* | 21.66 ± 1.77 | 17.88 ± 1.22 | 12.69 ± 1.03 | 9.77 ± 0.82 | 7.55 ± 0.55 | 0.00 ± 0.00 | 0.00 ± 0.00 | 32.55 ± 1.23 | 30.22 ± 1.18 | 25.33 ± 1.08 | 18.22 ± 1.11 | 15.66 ± 1.08 | 6.44 ± 0.62 | 0.00 ± 0.00 |
| *E. coli* | 29.66 ± 2.22 | 25.31± 1.83 | 21.71 ± 1.33 | 20.11 ± 1.16 | 13.59 ± 1.05 | 8.33 ± 0.73 | 0.00 ± 0.00 | 32.68 ± 1.12 | 30.58 ± 1.19 | 27.72 ± 1.21 | 13.66 ± 1.07 | 8.66 ± 0.73 | 6.22 ± 0.61 | 0.00 ± 0.00 |
| *K. pneumoniae* | 29.72 ± 1.88 | 24.51 ± 1.62 | 20.62 ± 1.26 | 15.29 ± 1.06 | 10.66 ± 0.94 | 7.88 ± 0.53 | 0.00 ± 0.00 | 30.53 ± 1.38 | 22.63 ± 1.25 | 16.77 ± 1.14 | 9.56 ± 0.63 | 0.00 ± 0.00 | 0.00 ± 0.00 | 0.00 ± 0.00 |
| *P. aeruginosa* | 24.74 ± 1.52 | 18.34 ± 1.35 | 14.44 ± 1.17 | 10.88 ± 1.05 | 8.11 ± 0.82 | 0.00 ±0.00 | 0.00 ± 0.00 | 33.39 ± 1.41 | 29.22 ± 1.32 | 27.34 ± 1.22 | 23.66 ± 1.27 | 19.29 ± 1.16 | 12.67 ± 1.06 | 7.81 ± 0.72 |

**Table S5** The antibacterial characteristics of the hydroethanolic extract of dried Roselle calyces against selective MDR bacterial clinical isolates compared with TGC as a reference standard antibiotic

| Antibacterial measurements | Roselle | | | | TGC | | | |
| --- | --- | --- | --- | --- | --- | --- | --- | --- |
|  | *A. baumanii* | *E. coli* | *K. pneumoniae* | *P. aeruginosa* | *A. baumanii* | *E. coli* | *K. pneumoniae* | *P. aeruginosa* |
| MIC (mg/mL) | 1.00 ± 0.123 | 0.50 ± 0.051 | 0.50 ± 0.038 | 1.00 ± 0.106 | 0.04 ± 0.009 | 0.20 ± 0.023 | 0.20 ± 0.031 | 0.02 ± 0.005 |
| MBC (mg/mL) | 1.25 ± 0.127 | 0.75 ± 0.034 | 0.75 ± 0.051 | 1.25 ± 0.161 | 0.10 ± 0.022 | 0.20 ± 0.038 | 0.40 ± 0.045 | 0.03 ± 0.007 |
| MBC/MIC | 1.25 | 1.50 | 1.50 | 1.25 | 2.50 | 1.00 | 2.00 | 1.50 |

Data values are expressed as means ± S.D. (n = 3).
